# The transition from HIF-1 to HIF-2 during prolonged hypoxia results from reactivation of PHDs and *HIF1A* mRNA instability

**DOI:** 10.1186/s11658-022-00408-7

**Published:** 2022-12-08

**Authors:** Maciej Jaśkiewicz, Adrianna Moszyńska, Jarosław Króliczewski, Aleksandra Cabaj, Sylwia Bartoszewska, Agata Charzyńska, Magda Gebert, Michał Dąbrowski, James F. Collawn, Rafal Bartoszewski

**Affiliations:** 1 International Research Agenda 3P- Medicine Laboratory, Medical University of Gdansk, Gdansk, Poland; 2 Department of Biology and Pharmaceutical Botany, Medical University of Gdansk, Gdansk, Poland; 3 Laboratory of Bioinformatics, Nencki Institute of Experimental Biology of the Polish Academy of Sciences, Warsaw, Poland; 4 Department of Inorganic Chemistry, Medical University of Gdansk, Gdansk, Poland; 5 Department of Cell, Developmental, and Integrative Biology, University of Alabama at Birmingham, BirminghamBirmingham, AL 35233 USA; 6 Department of Biophysics, Faculty of Biotechnology, University of Wroclaw, F. Joliot-Curie 14a Street, 50-383 Wroclaw, Poland

**Keywords:** Hypoxia, Human endothelial cells, *HIF1A*, *EPAS1*, HIF-1α, HIF-2α

## Abstract

The hypoxia-inducible factors (HIF) are transcription factors that activate the adaptive hypoxic response when oxygen levels are low. The HIF transcriptional program increases oxygen delivery by inducing angiogenesis and by promoting metabolic reprograming that favors glycolysis. The two major HIFs, HIF-1 and HIF-2, mediate this response during prolonged hypoxia in an overlapping and sequential fashion that is referred to as the HIF switch. Both HIF proteins consist of an unstable alpha chain and a stable beta chain. The instability of the alpha chains is mediated by prolyl hydroxylase (PHD) activity during normoxic conditions, which leads to ubiquitination and proteasomal degradation of the alpha chains. During normoxic conditions, very little HIF-1 or HIF-2 alpha–beta dimers are present because of PHD activity. During hypoxia, however, PHD activity is suppressed, and HIF dimers are stable. Here we demonstrate that HIF-1 expression is maximal after 4 h of hypoxia in primary endothelial cells and then is dramatically reduced by 8 h. In contrast, HIF-2 is maximal at 8 h and remains elevated up to 24 h. There are differences in the HIF-1 and HIF-2 transcriptional profiles, and therefore understanding how the transition between them occurs is important and not clearly understood. Here we demonstrate that the HIF-1 to HIF-2 transition during prolonged hypoxia is mediated by two mechanisms: (1) the HIF-1 driven increase in the glycolytic pathways that reactivates PHD activity and (2) the much less stable mRNA levels of HIF-1α (*HIF1A*) compared to HIF-2α (*EPAS1*) mRNA. We also demonstrate that the alpha mRNA levels directly correlate to the relative alpha protein levels, and therefore to the more stable HIF-2 expression during prolonged hypoxia.

**Supplementary Information:**

The online version contains supplementary material available at 10.1186/s11658-022-00408-7.

## Background

Cells that are unable to satisfy their physiological oxygen demand initiate the adaptive hypoxic response that stimulates angiogenesis, erythropoietin production, and metabolism reprogramming that favors glycolysis [1]. The hypoxia-inducible transcription factors (HIF-1 and HIF-2) interact with hypoxic-response elements (HREs) in the promoters and enhancers of their numerous target genes and play a major role in the transcriptional activation of the adaptive hypoxic response [2–4]. Transcriptionally active HIFs are heterodimers that assemble during times of hypoxia when the alpha subunit protein is dramatically stabilized [5]. During normoxia, HIF-α subunits undergo rapid prolyl hydroxylation by specific oxygen-dependent HIF prolyl-hydroxylases (PHDs) [6]. This prolyl hydroxylation results in their recruitment by von Hippel–Lindau tumor suppressor protein which is a component of an E3 ubiquitin ligase complex [7] and the resulting ubiquitination of the alpha subunit leads to proteasomal degradation [7, 8]. At low oxygen levels, PHD hydroxylation is inhibited and the HIF-α subunits accumulate and heterodimerize with the β subunits to form transcriptionally active HIF complexes [9].

HIF-1 complexes induce the expression of glycolytic genes [10], some pro-angiogenic genes, as well as genes involved in pH regulation [11]. HIF-2α that is encoded by *EPAS1* gene is expressed in specific cell types including endothelial cells (ECs), cardiomyocytes, and hepatocytes [12], and facilitates expression of matrix metalloproteinases and erythropoietin expression (13). Although it has been shown that angiogenesis is HIF-1-initiated, HIF-2 is required for the proper maturation of the vascular network [4]. Besides the unique HIF-1 and HIF-2 transcriptional targets, these factors also control the expression of a large set of overlapping genes [14]. Notably, in both cancer cells and ECs, HIF-1α accumulates earlier during hypoxia and its levels decrease more rapidly than HIF-2α during prolonged hypoxia [3, 4, 15, 16]. This results in a transition from HIF-1 to HIF-2 specific effects is called the HIF switch [17]. Although numerous factors have been proposed to contribute to the HIF switch [15, 18, 19] (reviewed in [17]), the mechanism underlying the HIF-1α elimination during prolonged hypoxia remains poorly understood. In earlier work, we found that the switch from HIF-1 to HIF-2 constitutes a universal mechanism of human endothelium adaptation to prolonged hypoxia [3]. We also observed that *HIF1A* mRNA stability is dramatically lower when compared to *EPAS1* mRNA during hypoxia [3]. However, although these studies suggested differences in these transcript stabilities as a potential key to understand the HIF switch, they did not provide a mechanistic explanation for the transition from HIF-1 to HIF-2 at the protein level.

A series of elegant studies demonstrated that in hypoxia exposed cells, nitric oxide and other respiratory chain inhibitors reduce mitochondrial oxygen demand, and this leads to increases in cytosolic oxygen and subsequent increases in PHD-dependent HIF-1α degradation [20–23]. These studies analyzed the effects of the redistribution of oxygen on HIF-1α only, however, and therefore the question remains how this mechanism relates to HIF-2 stability. Given that during hypoxia the HIF-1 activity promotes anaerobic glycolysis, and this further reduces mitochondrial oxygen consumption, this metabolic change could potentially reactivate PHDs [20–22]. Furthermore, different PHDs have different specificities on the HIF-α isoforms as well as different oxygen requirements, and the contribution of each PHD is related to its abundance [24]. For example, in human aortic endothelial cells during normoxia, the mRNA expression of *PHD3* is 2 times higher than that of *PHD2*, and 10 times higher than that of *PHD1* [24]. Furthermore, the expression of *PHD3* in endothelial cells is strongly induced during hypoxia, whereas the expression of *PHD1* and *2* remain relatively unchanged [25]. PHD3 has been shown to have comparable activity towards HIF-1α and HIF-2α [24]. It is therefore likely that the oxygen redistribution dependent reactivation of PHDs during hypoxia also affects the HIF-2α expression and thus could contributes to the HIF-switch. To our knowledge, the data on the effect of PHD reactivation on the activity of HIF-2 and the switch from HIF-1 to HIF-2 during the response to hypoxia have not been reported.

Herein, we report that during prolonged hypoxia in endothelial cells, the HIF-1/HIF-2 transition is a consequence of both restored PHDs activity and more stable *EPAS1* mRNA levels.

## Methods

### Cell culture

Primary human umbilical vein endothelial cells (HUVEC) that were pooled from ten individual donors, were purchased from Cellworks (cat. no. ZHC-2301, Caltag Medsystems Ltd, UK) and cultured in EGM-2 Bulletkit Medium (Lonza). At least three independent batches of HUVECs were used in the experiments. All experiments were conducted between passages 2 and 6 at a confluence of 70–80%.

### Induction of hypoxia

Hypoxia was induced in a CO_2_/O_2_ incubator/chamber specific for hypoxia research (Invivo2 Baker Ruskin). Briefly, cells were cultured in 35 mm or 60 mm dishes (for RNA isolation and protein isolation respectively) at 1% O_2_ for the time periods specified (PO_2_ was 10–12 mm Hg). Control cells were maintained in normoxia in a CO_2_/O_2_ incubator (Binder).

### Isolation of RNA

Total RNA (containing both mRNA and miRNA) was isolated using miRNeasy kit (Qiagen). RNA concentrations were calculated based on the absorbance at 260 nm. RNA samples were stored at − 70 °C until use.

### Measurement of mRNA quantitative Real Time PCR (qRT-PCR)

We used TaqMan One-Step RT-PCR Master Mix Reagents (Applied Biosystems) as described previously [26, 27] using the manufacturer’s protocol (retrotranscription: 15 min, 48 °C). The relative expressions were calculated using the 2^−ΔΔCt^ method [28] with the *RPLP0* gene as reference genes for the mRNA. TaqMan probes ids used were: *HIF1A* (Hs00153153_m1); *EPAS1 *(Hs01026149_m1); (Hs00420895_gH).

### Western blots

Cells were lysed in SDS lysis buffer (4% SDS, 20% glycerol, 125 mM Tris–HCl pH = 6.8) supplemented with protease inhibitors (cOmplete ™ Mini (Roche)). The insoluble material was removed by centrifugation at 15,000g for 15 min. Protein concentrations were determined by Bio-Rad™ DC-Protein Assay using bovine serum albumin (BSA) as standard. Following the normalization of protein concentrations, the lysates were mixed with an equal volume of 6X Laemmli sample buffer and incubated for 5 min at 95 °C prior to separation by SDS PAGE on Criterion TGX stain-free 4–15% gradient gels (Bio-Rad). Following SDS-PAGE, the proteins were transferred to polyvinylidene fluoride membranes (300 mA 4 h at 4 °C). The membranes were then blocked with BSA (Sigma-Aldrich) dissolved in PBS/Tween-20 (3% BSA, 0.5% Tween-20 for 1–2 h), followed by immunoblotting with the primary antibody: mouse anti-HIF-1α (1:2000, ab16066; Abcam); rabbit anti-HIF-2α (1:1000, ab199, Abcam); rabbit anti-β-Actin (1:1000, ab1801; Abcam). After the washing steps, the membranes were incubated with goat anti-rabbit IgG (H + L chains) or with goat anti-mouse IgG (H + L) HRP-conjugated secondary antibodies (Bio-Rad) and detected using SuperSignal West Pico ECL (Thermo Fisher Scientific). Densitometry was performed using Image Lab software v. 4.1 (Bio-Rad).

### Monitoring prolyl hydroxylase activity

To monitor prolyl hydroxylase-dependent degradation of Hif-α, we used reporter vector based on pEZX-FR02 cloning vector (GeneCopoeia) that contained *HIF1A* (NM_181054) or *EPAS 1* (NM_001430) gene region comprising oxygen-dependent degradation domain (ODD), amino acids 401–653 or 403–607, respectively, fused with Firefly luciferase (without the ATG start codon) in-frame downstream of the transgene and Renilla luciferase expressed from the same type but from an independent promoter as a transfection control [29–31]. As a control, unmodified pEZX-FR02 vector was used. Briefly, 24 h after transfection, the cells were seeded onto 96-well luminescence assay white plates with clear bottoms (Corning Inc., 3903) [29–31]. The next day the cells were exposed to hypoxia for indicated time points or to Dimethyloxalylglycine (DMOG, Sigma, D3695), and luciferase activity measured with Dual-Luciferase Reporter Assay System (Promega) in accordance with the manufacturer’s instructions (GloMax-Multi + Detection System (Promega)). Each time point was assayed in 3 technical replicates, experiments were repeated in 5 biological replicates. For each experimental condition, the control vector was used to calculate the fold changes.

### Statistical analysis

Results were expressed as a mean ± standard deviation. Statistical significance was determined using the Student’s t-test with P < 0.05 considered significant. Only pairwise comparisons were performed. The normality was tested with the Shapiro Wilk test. The correlation was accessed via the Pearson product-moment correlation method.

## Results

**Fig. 1:**
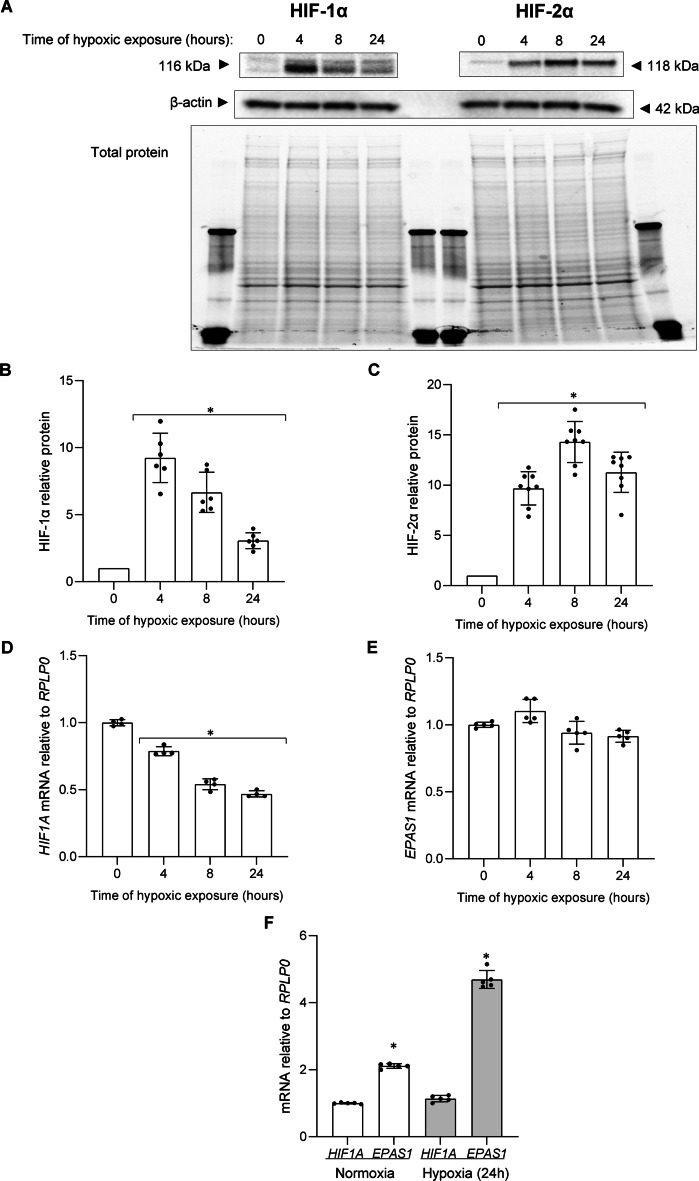
Hypoxia results in the accumulation of HIF-1α and HIF-2α in HUVECs. Cells were exposed to hypoxia for the time periods specified, and total RNA and protein lysates were collected. The changes in HIF-1α and HIF-2α protein levels were evaluated by western blot (**A**) normalized to β-actin and total protein levels and related to the normoxic control (**B**, **C**). Data represent the mean ± SD of at least four independent experiments. * P < 0.05 was considered significant. *HIF1A* and *EPAS1* mRNA levels were quantified by qRT-PCR and normalized to *RPLP0* mRNA levels, and expressed as a fold change over normoxic samples (**D**, **E**). Data represent the mean ± SD of at least four independent experiments (3 replicates each). * P < 0.05 was considered significant. The ratios between *HIF1A* and *EPAS1* mRNAs in HUVECs cultured in normoxia and hypoxia, based on qRT-PCR quantification in the same samples, the *HIF1A* mRNA level was considered as one for both conditions (**F**). Data represent the mean ± SD of five independent experiments (3 replicates each). * P < 0.05 was considered significant

We previously reported that during hypoxia in human ECs HIF-1 is eventually replaced by the HIF-2 [3, 15, 17], and our goal here was to explore the underlying mechanism. As shown in Fig. 1A**,** we followed the HIF-1α and HIF-2α protein levels in human umbilical vein endothelial cells (HUVECs) at 4-, 8- and 24-h exposure to hypoxia (1% O_2_). Consistent with our previous findings [15, 26, 27], we observed that HIF-1α rapidly accumulated in HUVECs exposed to hypoxia at 4 h and was reduced at 8 h and dramatically reduced at 24 h. In contrast, HIF-2α reached maximal levels at 8 h, and remained elevated even after 24 h. Hence, although in ECs both HIF-1α and HIF-2α at first rapidly accumulate during hypoxia, the rapid loss of HIF-1 levels leads to the transitional switch to HIF-2 signaling. The hypoxic changes of mRNA and protein levels for both *HIF1A*/HIF-1α and *EPAS1*/HIF-2α were well correlated (Fig. 1B–D). Interestingly, the hypoxic accumulation of HIF-1α subunits was also accompanied by the continuous decline in *HIF1A* mRNA (Fig. 1D), whereas *EPAS1* mRNA levels remained fairly constant during studied time course (Fig. 1E). Furthermore, *EPAS1* mRNA levels are two-fold higher than *HIF1A* in normoxia and five-fold higher during hypoxia at 24 h (Fig. 1F).

**Fig. 2:**
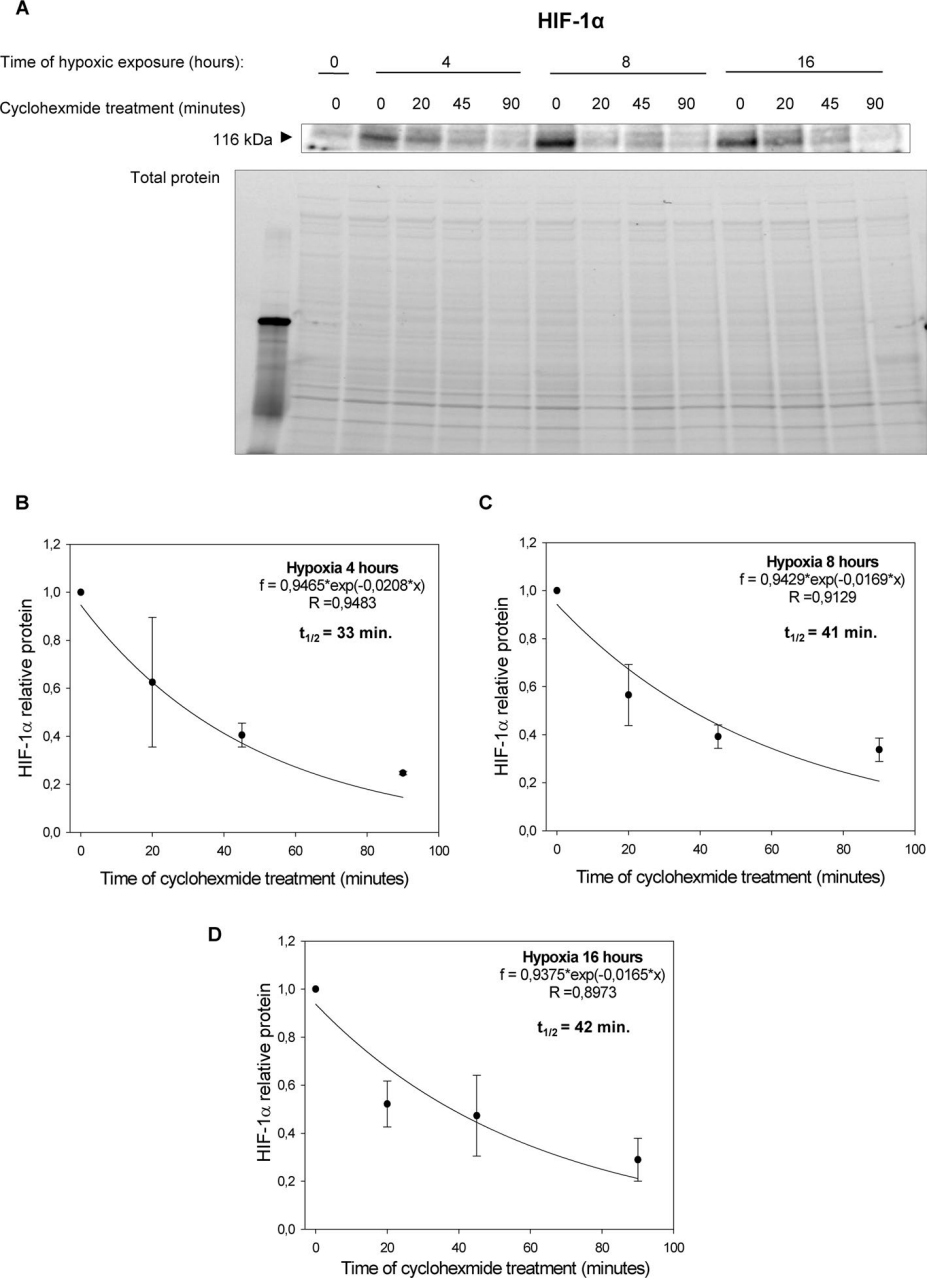
Hypoxia effect on HIF-1α protein half-live. **A** HIF-1α half-live measurements were taken in HUVECs exposed to hypoxia for 4, 8, and 16 h (cells cultured in normoxia were the negative control). Cycloheximide was added to stop translation, after which protein lysates were collected, and HIF-1α levels at each time point were measured by western blot and normalized to total protein levels. Values for each time point were calculated from three individual samples generated in at least three independent experiments. The mathematical representation of HIF-1α protein levels for 4 h (**B**), 8 h (**C**) and 16 (**D**) hypoxia in HUVECs. The time points indicating relative HIF-1α levels were plotted as differences from *HIF-1α* levels at the initial time point (t = 0). The protein half-lives were calculated from the exponential decay using the trend line equation C/C_0_ = e^–kdt^ (where C and C_0_ are protein amounts at time t and at the t_0_, respectively, and k_d_ is the protein decay constant). The error bars represent SD. * P < 0.05 was considered significant

**Fig. 3:**
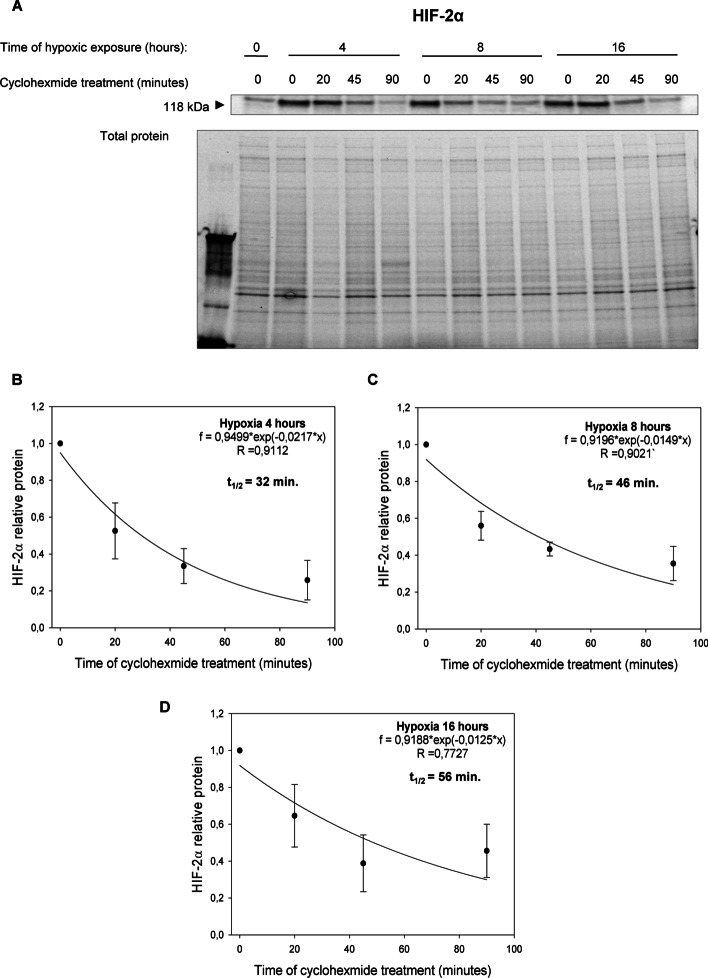
Hypoxia effect on HIF-2α protein half-live. **A** HIF-2α half-live measurements were taken in HUVECs exposed to hypoxia for 4, 8, and 16 h (cells cultured in normoxia were negative control). Cycloheximide was added to stop translation, after which protein lysates were collected, and HIF-2α levels at each time point were measured by western blot and normalized to total protein levels. Values for each time point were calculated from three individual samples generated in at least three independent experiments. The mathematical representation of HIF-2α protein levels for 4 h (**B**), 8 h (**C**) and 16 (**D**) hypoxia in HUVECs. The time points indicating relative HIF-1α levels were plotted as differences from *HIF-2α* levels at the initial time point (t = 0). The protein half-lives were calculated from the exponential decay using the trend line equation C/C_0_ = e^–kdt^ (where C and C_0_ are protein amounts at time t and at the t_0_, respectively, and k_d_ is the protein decay constant). The error bars represent SD. * P < 0.05 was considered significant

Surprisingly, however, in cells cultured in hypoxia HIF-1α and HIF-2α protein half-lives were comparable after 4- and 8-h exposure to hypoxia (Figs. 2 and 3): HIF-1α ^4 h hypoxia^ t_1/2 ≈_ 33 min and HIF-2α ^4 h hypoxia^ t_1/2 ≈_ 32 min; HIF-1α ^8 h hypoxia^ t_1/2 ≈_ 41 min and HIF-2α ^8 h hypoxia^ t_1/2 ≈_ 46 min. Whereas after 16 h of exposure to hypoxia HIF-2α was slightly more stable than HIF-1: HIF-1α ^16 h hypoxia^ t_1/2 ≈_ 42 min and HIF-2α ^16 h hypoxia^ t_1/2 ≈_ 56 min. Notably, the HIF-1α protein half-live measured in HUVECs exposed to hypoxia for 4 h is consistent with the previous report of Hagen et al. [20], whereas to the best of our knowledge, no data regarding other times regarding the half-live HIF-2α protein stability during hypoxia are available, although some data from HIF-1 and HIF-2 overexpression were presented previously [32]. These results suggest that the hypoxic transition from HIF-1 to HIF-2 is a result of decreased *HIF1A* mRNA levels rather than the small differences in HIF-1α and HIF-2α subunits protein stability at the later time points during hypoxia.

Since, the oxygen-dependent activity of PHDs provides a canonical mechanism of HIF-α subunits destabilization in normoxia, and PHDs (PHD-2 and PHD-3) were shown to have different preference against the HIF-1α and HIF-2α subunits as well as oxygen requirements [13, 33], we tested whether the observed earlier degradation of HIF-1α might also result from reactivation of these enzymes during prolonged hypoxia. Given that during hypoxia HIF-1 activity coincides with an activation of anaerobic glycolysis and inhibition of the mitochondrial aerobic metabolism, we tested whether these changes could lead to reactivation of PHDs [20, 34].

**Fig. 4:**
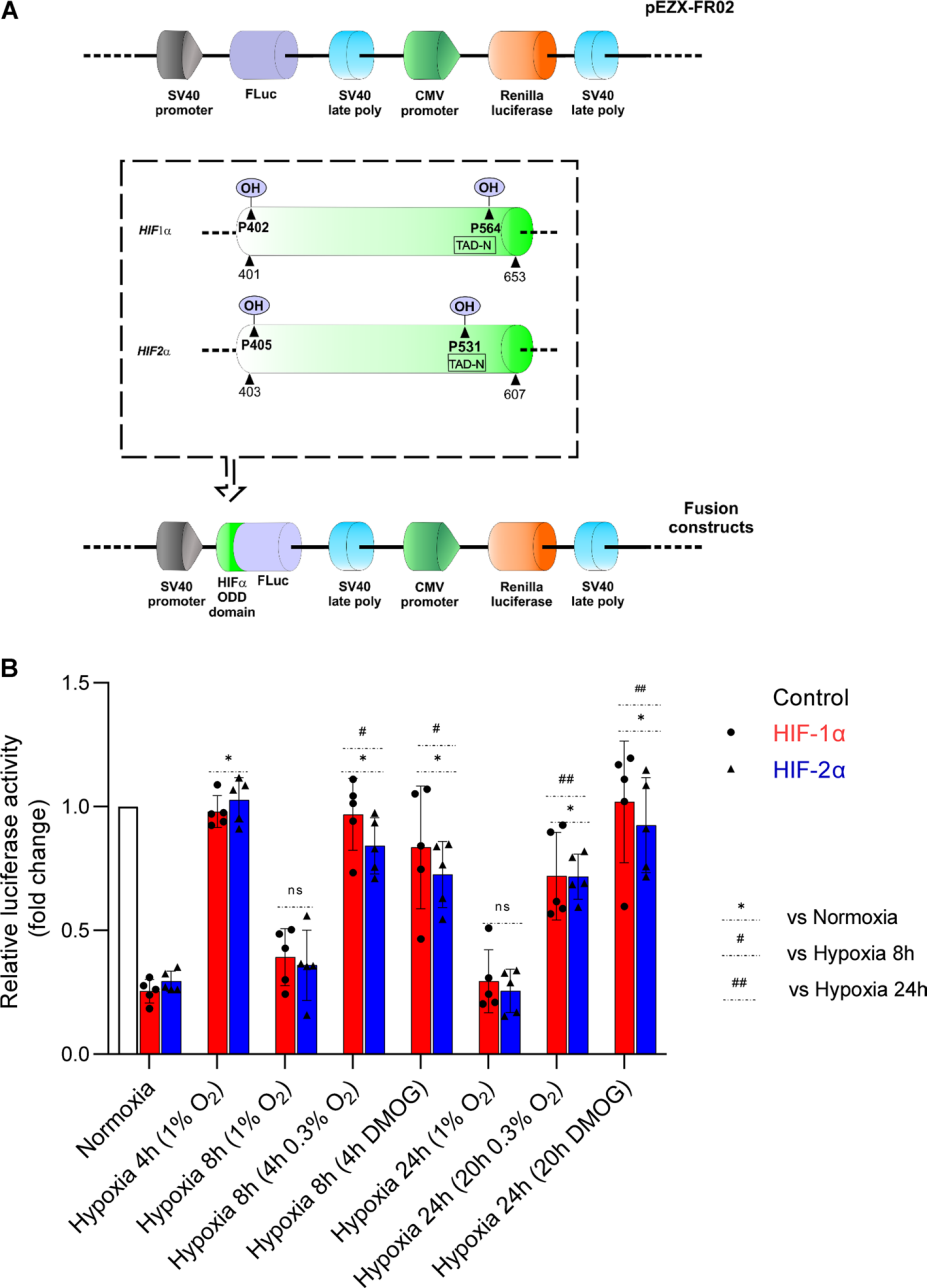
Prolyl hydroxylase inactivation in HUVECs influences ODD domains stability during hypoxia. **A** Schematic presentation of firefly luciferase (FLuc) and renilla luciferase reporter cloning vector pEZX-FR02 (GeneCopoeia) containing the *HIF1A* human (NM_181054) gene region comprising ODD (amino acids 401–653, including the Prolines 402 and 564 (a PHD substrate) or human *EPAS1* (NM_001430) gene region comprising ODD (amino acids 403–607, including the Prolines 404 and 531 (a PHD substrate). These gene regions were fused with firefly luciferase (without the ATG start codon) downstream of the *HIF-α*. **B** Hypoxia-related stabilization of the HIF-1α and HIF-2 α ODD reporter. HUVECs following the transfection with the HIF-α-ODD reporter vectors were exposed to hypoxia (1% O_2_) for indicated time points in the presence or absence of 2.5 mM DMOG, or exposed to 0.3% O_2_ (both DMOG addition and the exposure to 0.3% O_2_ were performed after 4 h form the experiments start) and luciferase activity measured with Dual-Luciferase Reporter Assay System. The locations of the proline hydroxyl addition that destabilizes the HIF alpha subunits is illustrated in the panel A box. The firefly luciferase data were normalized to Renilla signal and expressed as a fold change over the control vector (without HIF-α-ODD) in the same experimental conditions. R.L.U. – relative light units. Data represent the mean ± SD of five independent experiments (3 replicates each). The P < 0.05 was considered significant and depicted as follows: * (for pairwise comparison to normoxia), # (for pairwise comparison to hypoxia 8 h), ## (for pairwise comparison to hypoxia 24 h)

To test this, we used Firefly luciferase reporter vectors (the Renilla luminescence was used as a calibrator of transfection efficiency) containing the respective HIF-1α and HIF-2α oxygen-dependent degradation domains (ODDs) and a control vector, lacking the ODD region, as shown in Fig. 4A [5, 35] and Additional file 1: Figure S1. The presence of HIF-1α and HIF-2α ODDs resulted in significantly reduced Firefly luminescence to less than one-third of the observed for control vector, when the transfected HUVECs were cultured in normoxia. Furthermore, as expected, when the transfected cells were incubated at 1% oxygen for 4 h, the presence of HIF-1α and HIF-2α ODD domains had no significant impact on the Firefly luminesce, that was comparable to the control vector, confirming that PHD-dependent degradation of both HIF-α subunits was inhibited. However, in cells that were incubated at 1% O_2_ for 8 h and 24 h, the Firefly luminescence from both HIF-1α and HIF-2α reporters was significantly reduced, when compared to signal measured after 4 h incubation at 1% O_2_ (Fig. 4B). Notably, the luminescence of HIF-1α and HIF-2α reporters after 8- and 24-h exposure to hypoxia was comparable to the one measured during normoxia, suggesting that at prolonged exposure to hypoxia PHD activity has been restored. Furthermore, HIF-1α and HIF-2α ODDs Firefly luminescence recorded for their respective reporter vectors were not significantly different between each other, neither in normoxia nor during the time course exposure to hypoxia (Fig. 4B), suggesting that PHD-dependent degradation has similar impact on both HIF-1α and HIF-2α ODD domains.

To confirm this result another way, we investigated effects of blocking PHD activity with dimethyloxalylglycine (DMOG), a competitive inhibitor of the endogenous 2-oxoglutarate cofactor for prolyl hydroxylase activity [36]. Following HUVECs transfection with luciferase control, HIF-1α ODD. and HIF-2α ODD reporter vectors, cells were incubated for 4 h at 1% O_2_ and then cells were incubated for next 4 h (total 8 h at 1% O_2_) and 20 h (total 24 h at 1% O_2_) in the absence or presence of 2.5 mM DMOG. As shown in Fig. 4B, during hypoxia DMOG prevented the reduction of Firefly luminescence from both HIF-1α ODD and HIF-2α ODD reporter vectors at 8 h and 24 h of hypoxia. Taken together, these results confirmed that HIF-α subunits degradation during prolonged hypoxia is due to PHD activity.

Given that PHD activity was present during prolonged hypoxia, in follow-up experiments we tested whether we could prevent of the restoration of cytosolic oxygen homeostasis by exposing HUVECs to 0.3% O_2_ and whether the PHDs would retain activity. In these experiments, the HUVECs were transfected with luciferase control, HIF-1α ODD, and HIF-2α ODD reporter vectors and were incubated for 4 h at 1% O_2_, and transferred to 0.3% O_2_ for another 4 h (total 8 h of hypoxia) or 20 h (total 24 h of hypoxia). The Firefly luminescence from both HIF-α ODD reporter vectors after 8 and 24 h remained at the levels observed when cells were exposed to 1% O_2_ for 4 h, and significantly higher than in cells incubated at 1% O_2_ for the same time (Fig. 4B). These results support the hypothesis that the restoration of PHDs activity during prolonged hypoxia exposure results from the recovery of cellular oxygen levels due to adaptive hypoxia response related to the reduction of mitochondrial oxygen consumption.

**Fig. 5:**
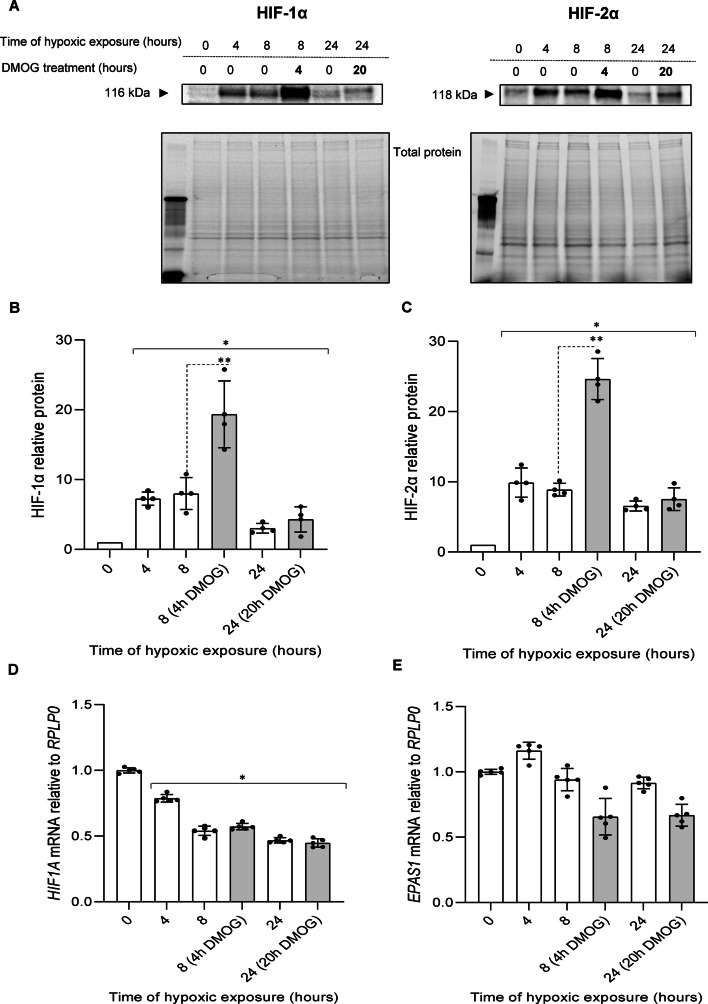
Inhibition of PHDs activity during hypoxia with DMOG results in accumulation of HIF-1α and HIF-2α in HUVECs. Cells were exposed to hypoxia in the presence or absence of 2.5 mM DMOG that was added (grey bars) after 4 h from the experiments start and for the time periods specified, the total RNA and protein lysates were collected. The changes in HIF-1α and HIF-2α protein levels were evaluated by western blot (**A**) normalized to total protein levels and related to the normoxic control (**B**, **C**). Data represent the mean ± SD of four independent experiments * P < 0.05 was considered significant. *HIF1A* and *EPAS1* mRNA levels were quantified by qRT-PCR and normalized to *RPLP0* mRNA levels and expressed as a fold change over normoxic samples (**D**, **E**). Data represent the mean ± SD of five independent experiments (3 replicates each). * P < 0.05 was considered significant

To confirm the validity of the luciferase results, we tested blocking the PHD activity during hypoxia with DMOG on the HIF-1α and HIF-2α protein levels. As shown in Fig. 5A, B, when HUVECs were exposed to hypoxia for 4 h, and then DMOG was added, and cells were cultured in hypoxic conditions for another 4 h (total time of hypoxic exposure was 8 h), the HIF-1α levels were dramatically (about 2 times) higher than in HUVECs exposed to hypoxia for 8 h without the DMOG addition. Similar, DMOG-related rescue of α subunit protein was also observed in parallel HIF-2α experiments (Fig. 5A, C). The HIF-2α levels doubled after 8 h of exposure to hypoxia if the DMOG was added after 4 h. These DMOG-induced HIF-1α and HIF-2α protein levels during hypoxia are consistent with the luciferase reporters results and strongly supports the possibility of the hypoxic reactivation of PHDs during prolonged hypoxia. However, in contrast to luciferase reporter results, no significant HIF-1α and HIF-2α accumulation in the DMOG presence was observed after 24 h exposure to hypoxia (Fig. 5A–C). Considering that these DMOG treatments did not significantly affected *HIF1A* and *EPAS1* mRNA levels (Fig. 5D, E), this data suggest that during prolonged hypoxia, PHDs are active and account for both HIF-1α and HIF-2α degradation. Notably, however, although DMOG has been reported as an effective PHDs activity inhibitor, it has pleiotropic effects on the cellular metabolism and different effects on HIF-1α and HIF-2α have been reported for this hypoxia mimetic [36]. It has also been shown that treatment with DMOG, can result in the accumulation of HIF-1α but not of HIF-2α [37]. Hence, although the expected rescue of HIF-2α protein was not observed in 24 h DMOG experiments, despite the relatively high *EPAS1* mRNA levels, this result needed to be confirmed via another approach.

**Fig. 6:**
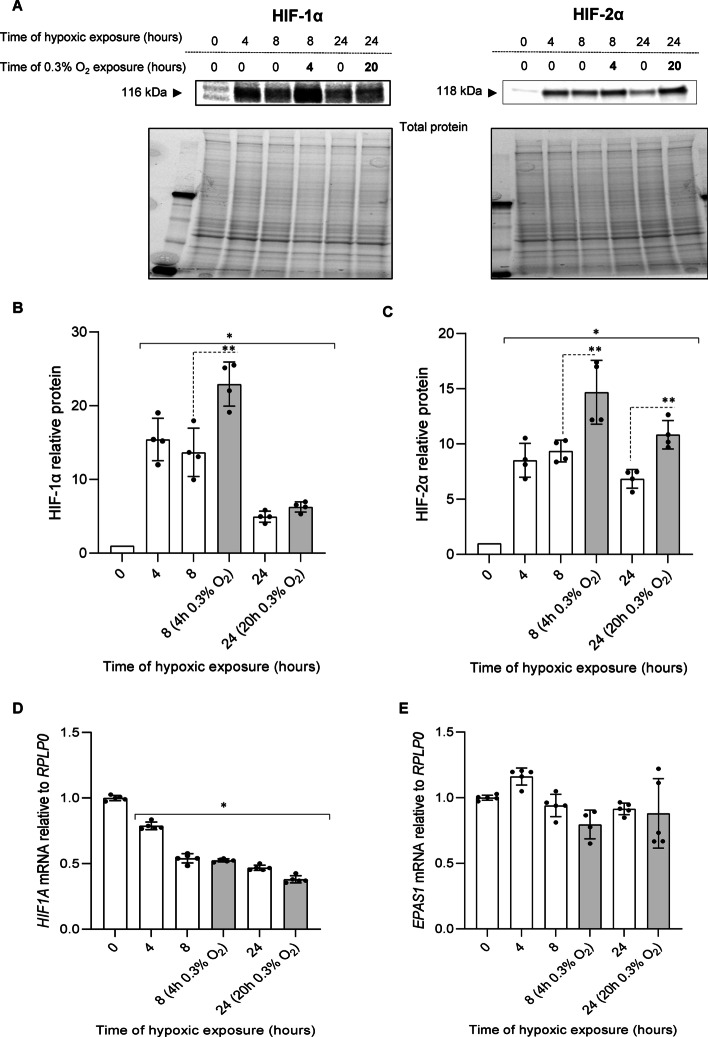
Inhibition of PHD activity during hypoxia by reducing oxygen levels results in accumulation of HIF-1α and HIF-2α in HUVECs. Cells were exposed to 1% O_2_ for 4 h, and next moved to 0.3% O_2_ (grey bars) or remained in the same conditions for the time periods specified, and total RNA and protein lysates were collected. The changes in HIF-1α and HIF-2α protein levels were evaluated by western blot (**A**) normalized to total protein levels and related to the normoxic control (**B**, **C**). Data represent the mean ± SD of four independent experiments. * P < 0.05 was considered significant. *HIF1A* and *EPAS1* mRNA levels were quantified by qRT-PCR and normalized to *RPLP0* mRNA levels and expressed as a fold change over normoxic samples (**D**, **E**). Data represent the mean ± SD of five independent experiments (3 replicates each). * P < 0.05 was considered significant

As an alternative approach, HUVECs were exposed to hypoxia (1% O_2_) for 4 h and then O_2_ was reduced to 0.3%, and cells remained in these conditions for another 4 h (total time of hypoxic exposure was 8 h). For the 24-h time point, the cells were changed to 0.3% oxygen for 20 h. The results were interesting in that in the 8-h time point, the HIF-1α levels were increased when incubated in the 0.3% oxygen for 4 h (Fig. 6A, B), and the HIF-2α was also affected under the same conditions (Fig. 6A, C). At the 24-h time point the HIF-2α protein level was significantly elevated in the low oxygen, whereas the HIF-1α protein levels were not (Figure A, C). Furthermore, the reduced oxygen levels had no significant impact on *HIF1A* nor *EPAS1* expression (Fig. 6D, E). Taken together, HIF-α subunits rescue observed upon further reduction of oxygen availability (0.3%) during hypoxia strongly supports some PHD activity at 1% oxygen caused by the cellular oxygen redistribution potentially mediated by HIF-1 and HIF-2. Importantly, the data support the hypothesis that the inability to effectively rescue HIF-1α during prolonged hypoxia is mediated by both PHD activity and mRNA instability.

During hypoxia, initially both HIF-1α and HIF-2α accumulate due to the reduced PHDs activity. The NO-mediated inhibition of the respiratory chain and metabolic switch to glycolysis, however, results in lower mitochondrial oxygen consumption and leads to restoration of some cellular O_2_ that is sufficient for PHDs-related degradation of both of these HIF-α subunits [20–23]. Notably, however, the *HIF1A* mRNA levels are also reduced during hypoxia, limiting the amount of translated HIF-1α, whereas the *EPAS1* mRNA is more abundant and much more stable, allowing for the continuous accumulation of HIF-2α during prolonged hypoxia.

Notably, we were able to simulate the effects of changing oxygen concentration and of the reduction of *HIF1A* transcript in the time-course of hypoxia using a dynamic model, in which the effect of the oxygen level on the hydroxylation of the two HIF-α subunits by PHDs was modeled according to an established dynamic model of HIF-1 signaling [38]. In our model, we assumed that the system contains a single dominant PHD isoform with potentially different activities towards HIF-1α and HIF-2α, in agreement with the experimental evidence for ECs [24, 25]. The effects of transcript abundancies of *HIF1A* and *EPAS* were modeled using the Mass-Action Law. This model was first fitted to time-series data of 12 time-points from HUVECs under 1% hypoxia. We then used the fitted model to assess (i.e., predict) the response to a further drop in the oxygen level to 0.3% at 4 h of hypoxia. For details of the modeling see Additional file 3. Our results clearly support the conclusion that the residual PHD activity during prolonged hypoxia together with the *HIF1A* mRNA instability contribute to the HIF-1α to HIF-2α transition during hypoxia.

## Discussion

Although changing HIF-α isoform dependency during hypoxia has been recognized both in endothelial and cancer cells [4, 17, 39, 40] as an important regulator of the adaptive response and a potential therapy target, the mechanisms responsible for the hypoxic HIF-1α protein destabilization under low oxygen pressure were less clear. Previous studies in cancer cells have identified hypoxia-associated factor (HAF), that selectively binds and degrades HIF-1α in an oxygen-dependent manner, whereas its binding to HIF-2α selectively increases this factor transactivation [41]. HAF has been reported to be overexpressed in tumors, whereas its expression in cancer cells is reduced during acute hypoxia and elevated during prolonged hypoxia, providing an elegant explanation for the tumor HIF1/HIF2 transition [41]. Despite undisputable function in cancer models, HAF expression in human endothelial cells is not significantly affected by hypoxia [3] (Additional file 2: Figure S2). To our knowledge, there are no reports of HAF-mediated HIF transition in human endothelium.

In our previous studies, we showed that the HIF switch constitutes a universal mechanism of cellular adaptation to hypoxia in human endothelial cells [3]. In the same study, we also observed that the hypoxic reduction of *HIF1A* mRNA was more dramatic than of *EPAS1* mRNA and this was attributed to the changes in mRNA half-lives, whereas similar results were also reported previously for some other ECs [42–44]. The higher stability of *EPAS1* mRNA could be the result of lower susceptibility to AU rich elements (ARE)-dependent transcript destabilization [45–48] as well, or that EPAS1 is less prone to miRNA-dependent degradation [3, 15, 17]. Notably, no miRNA has been proposed to directly degrade *EPAS1* mRNA in hypoxic ECs, whereas many *HIF1A* mRNA specific miRNAs have been identified [15]. Nevertheless, chronic hypoxia impairs microRNA biogenesis in a von Hippel-Lindau-dependent manner [49] and thus potentially the mRNA stability of both HIF-α isoforms. However, considering complexity of miRNA related networks, their impact on the HIF switch requires further study. Furthermore, as an increase in the natural antisense form of the HIF-1α transcript, *HIF1A-AS2*, expression during prolonged hypoxia has been also shown to reduce *HIF1A* expression [50, 51].

Although the differences in ARE- and miRNA- dependent degradation of *HIF1A* and *EPAS1* mRNAs can explain lower *HIF1A* mRNA half-life during hypoxia, these differences might be too small to be entirely responsible for the HIF-1 to HIF-2 switch. In this study, using different batches of HUVECs collected for 10 independent donors, we not only confirmed lower *HIF1A* mRNA levels in both normoxia and hypoxia when compared to the *EPAS1* mRNA, but we also accessed the ratio between these two transcripts. Notably, during prolonged hypoxia *EPAS1* mRNA level was about five-fold higher than that of *HIF1A* mRNA level, suggesting that this more limited mRNA availability may be the limiting factor for the hypoxic HIF-1α accumulation. Furthermore, our analysis of HIF-1α and HIF-2α protein half-lives shown that these HIFα isoforms were comparably stable during both acute and prolonged hypoxia. Hence, selective destabilization of HIF-1α during prolonged hypoxia was not a plausible explanation of HIFs transition in this model. Importantly, both HIF-1α and HIF-2α protein half-lives during hypoxia are less than 1 h and that qualifies them as a short-lived proteins (below 8 h) [52]. This indicates that they are both subject to fairly rapid degradation even during hypoxic conditions. However, the canonical mechanism of HIF-mediated hypoxic responses assumes that these proteins accumulate during hypoxia since the lack of oxygen impairs their PHD-mediated posttranslational modifications and subsequent proteasomal degradation [53]. Importantly, PHDs have different specificities for the HIF-α isoforms as well as different oxygen requirements [24]. During a tumor’s adaptive response to acute hypoxia, increases in the vascular network increase oxygen delivery and restore the activity of PHD-2 and factor inhibiting HIF-1 (FIH-1) prevent HIF-1α accumulation and its related transcriptional activity [24, 54]. Whereas complete restoration of oxygen homeostasis reactivates PHD-3 and leads to HIF-2α elimination and loss of HIF signaling [24]. Despite their different affinities for HIF-α isoforms, PHDs when active can still effectively direct both HIF-1α and HIF-2α to proteasomal degradation. In our experimental model of hypoxia, oxygen was maintained at 1% through the entire time course, and thus oxygen homeostasis remained unrestored.

In the elegant study of Hagen and coworkers, the authors demonstrated that in hypoxia exposed ECs, the adaptive response related inhibition of mitochondrial respiration led to a cellular oxygen redistribution that restored prolyl hydroxylase activity [20]. Furthermore, they observed that inhibitors of mitochondrial respiration led to HIF-1α destabilization during hypoxia [20]. Here, we used a similar approach utilizing HIF-1α and HIF-2α ODD luciferase reporters and observed that although they are both stabilized during acute hypoxia, their signal levels return to those observed in normoxia after 8 h. Furthermore, chemical inhibition of prolyl hydroxylase activities with DMOG as well as preventing the reestablishment of cytosolic oxygen levels via transferring the cells to 0.3% oxygen increased the levels of HIF-1α ODD and HIF-2α ODD luciferase reporter signals during prolonged hypoxia. These data suggested that the redistribution of cellular oxygen during prolonged hypoxia caused by the adaptive response was related to the switch to glycolysis, and this was sufficient in reactivation of the PHD activities to decrease both HIF-1α and HIF-2α proteins. Notably, in these experiments we did not observe any significant difference between the HIF-1α and HIF-2α ODD luciferase reporter responses to hypoxia, 0.3% oxygen or DMOG, that could be associated with a PHD-2 or PHD-3 preference for these ODD domains of the two HIF-α isoforms. However, it has to be stressed that these reporters consist of the HIF-α ODD domains only and thus may not reflect the PHD selectivity against the entire HIF-α proteins.

To focus on the effects of the hypoxic reactivation of PHDs on the HIF-1/HIF-2 transition we performed parallel experiments in which we followed changes in HIF-1α and HIF-2α protein levels in hypoxia exposed HUVECs in the presence of DMOG as well as when the cells were transferred to 0.3% oxygen. The inhibition of PHDs resulted in spectacular increase of both HIF-1α and HIF-2α levels after eight of hours exposure to hypoxia but not after 24 h, only partially confirming the luciferase assays results. Since the DMOG treatments did not affect *HIF1A*, the lack of HIF-1α rescue in cells exposed to hypoxia for 24 h in this inhibitor presence was expected due to low *HIF1A* mRNA levels and short protein half-life. However, lack of DMOG-mediated HIF-2α rescue at 24 h’ time point was problematic since the *EPAS1* mRNA levels were not reduced, and this inhibitor in luciferase assays was effectively preventing HIF-2α-ODD domain degradation at this time point. Inability of DMOG to prevent HIF-2α destabilization during prolonged hypoxia is however in good agreement with previous reports showing that this compound treatment’s favors HIF-1α accumulation [36, 37]. Further studies are required to establish the mechanisms governing the DMOG-`related HIF-1α preference.

Importantly, however, when we prevented hypoxic reactivation of PHDs by reducing available oxygen levels, we observed higher accumulation HIF-2α but not HIF-1α at 24 h’ time point, consistent with luciferase reporters assays results. These findings are in very good agreement with reports of Hagen et al., and confirm functional reactivation of PHDs in prolonged hypoxia due to cellular oxygen redistribution [20]. Notably, transferring the cells to lower oxygen conditions (0.3%) did not affect mRNA levels of *HIF1A* and *EPAS1* compared to 1% oxygen.

## Conclusions

Taken together, our data show that during acute hypoxia HIF-1α and HIF-2α accumulate due to the impairment of PHDs activity, whereas during prolonged hypoxia, redistribution of cellular O_2_ leads to PHDs reactivation and consequently restarts HIF-α degradation. This begins to happen in our studies sometime after 8 h based on the levels of HIF-2α (Fig. 1). Hence, although other mechanisms responsible for selective degradation of the HIF-α during hypoxia cannot be excluded [18, 41], the HIF-α expression during prolonged hypoxia results from competition between *HIF1A* and *EPAS1* mRNA expression and the PHD-driven protein destabilization. Considering the comparable stability of HIF-1α and HIF-2α subunits, the lower levels of *HIF1A* mRNA as well as their further reduction during prolonged hypoxia result in faster elimination of HIF-1α from hypoxia exposed cells and thus promote transition of signaling from HIF-1 to HIF-2.

Furthermore, our data suggest that the HIF transition is a consequence of the adaptive response to hypoxia and results from reactivation of HIF-α degradation. Although further studies are necessary to establish the role of these mechanisms in vivo for regulating HIF signaling, we believe that our findings provide a basis for a novel definition of acute hypoxia as a condition preceding hypoxic reactivation of HIF-α degradation.

## Supplementary Information

**Additional file 1.** Figure S1.

**Additional file 2.** Figure S2.

**Additional file 3.** Supplementary Information 1.

**Additional file 4.** Supplementary File 1a.

**Additional file 5.** Supplementary File 2.

**Additional file 6.** Supplementary Materials - western blots.

## Data Availability

All data generated or analysed during this study are included in this published article (and its Additional files 4, 5, 6).

## Abbreviations

ARE: AU rich element
BSA: Bovine serum albumin
DMOG: Dimethyloxalylglycine
ECs: Endothelial cells
*EPAS1*: Hypoxia: inducible factor 2 alpha
FIH: 1: Factor: inhibiting hypoxia: inducible factor: 1
*GLUT1*: Glucose transporter 1
HAF: Hypoxia: associated factor
HIF: Hypoxia: inducible factor
HIF-α: Hypoxia-inducible factor alpha
HIF-1: Hypoxia-inducible factor 1
HIF-2: Hypoxia-inducible factor 2
HRE: Hypoxia response element
HUVEC: Human umbilical vein endothelial cells
id: Identification
miRNA: MicroRNA
NO: Nitric oxide
ODD: Oxygen dependent degradation domain
PBS: Phosphate buffer saline
PHD-2: Prolyl hydroxylase 2
PHD-3: Prolyl hydroxylase 3
PHDs: Prolyl: hydroxylases
qRT-PCR: Quantitative real-time PCR
SD: Standard deviations
SDS: Sodium dodecyl sulphate
SDS-PAGE: Sodium dodecyl sulphate–polyacrylamide gel electrophoresis
VEGFA: Vascular endothelial growth factor A
