# Supplementary figures and images for: The transition from HIF-1 to HIF-2 during prolonged hypoxia results from reactivation of PHDs and HIF1A mRNA instability
Source: Cell Mol Biol Lett. 2022 Dec 8;27:109. doi: 10.1186/s11658-022-00408-7 (PMC9730601; doi:10.1186/s11658-022-00408-7)

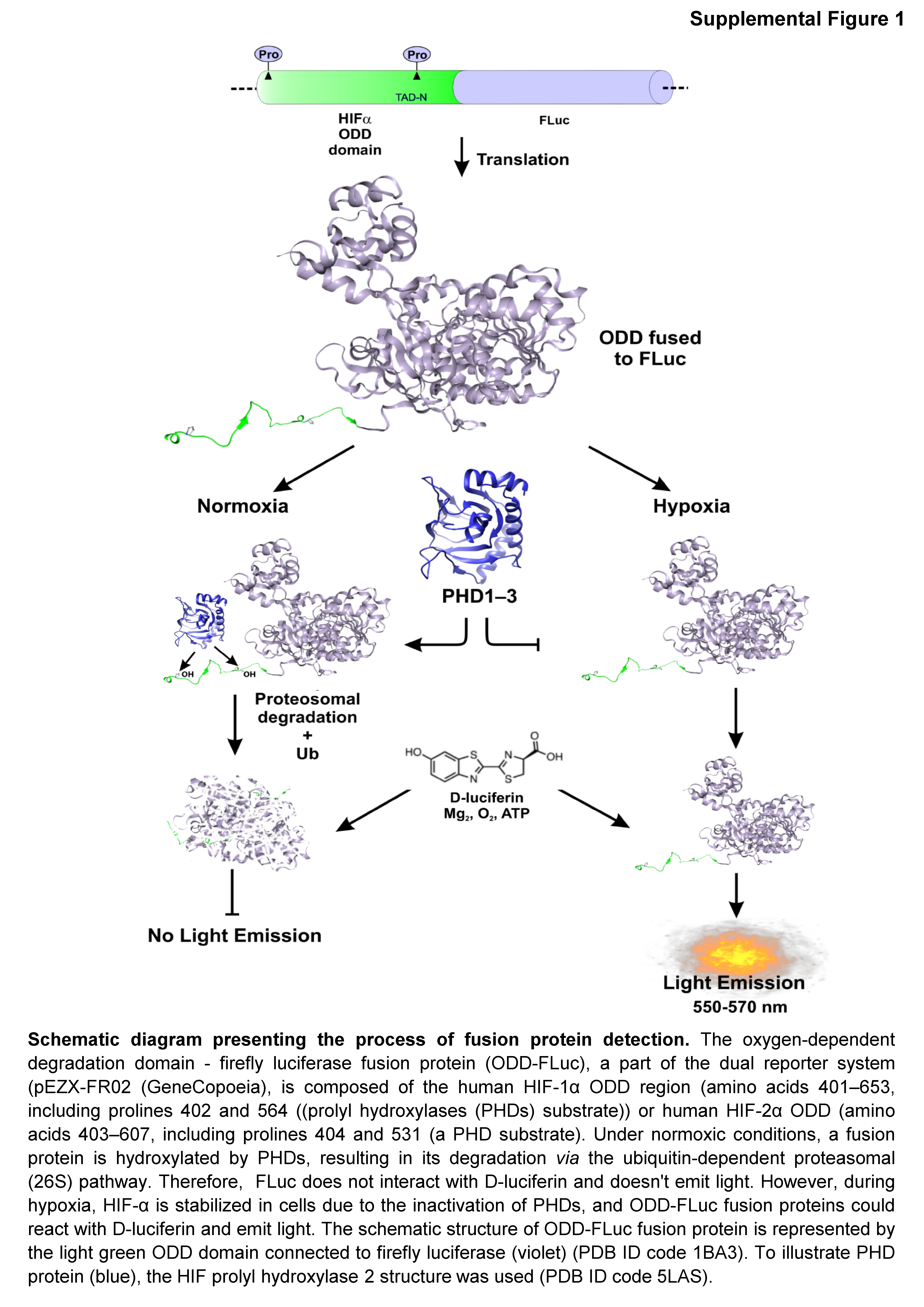

Supplement: Supplementary file 1 — Additional file 1. Figure S1. [file 11658_2022_408_MOESM1_ESM.tif]

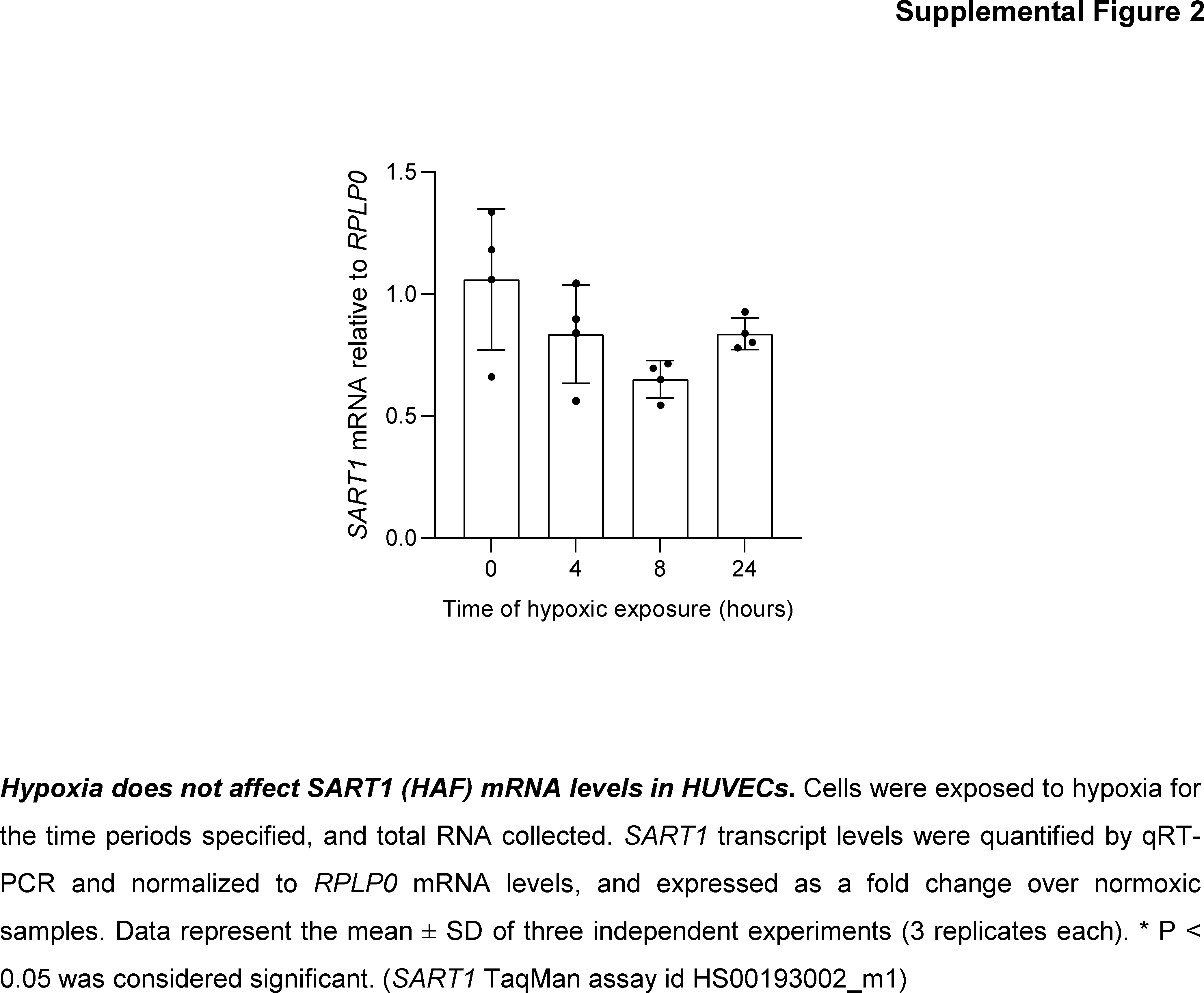

Supplement: Supplementary file 2 — Additional file 2. Figure S2. [file 11658_2022_408_MOESM2_ESM.tif]
