# Supplementary material for: The transition from HIF-1 to HIF-2 during prolonged hypoxia results from reactivation of PHDs and HIF1A mRNA instability
Source: Cell Mol Biol Lett. 2022 Dec 8;27:109. doi: 10.1186/s11658-022-00408-7 (PMC9730601; doi:10.1186/s11658-022-00408-7)
Supplement: Supplementary file 3 — Additional file 3. Supplementary Information 1. [file 11658_2022_408_MOESM3_ESM.pdf]

Supplementary Information 1

The cellular response to hypoxia by the accumulation of HIF-1α protein has been extensively modeled before with ordinary differential equations (ODEs) – reviewed in Cavadas et al.[1] (2013). In the current work, we implemented and verified a simple ODE model to assess the combined effects of changes in the oxygen level – affecting the hydoxylation of HIFs α by PHD, and of changing *HIF1A* and *EPAS1* transcripts levels – affecting HIFs α production, on the accumulation of HIF-1α and HIF-2α during hypoxia. In our model, the key equation for the rate of HIF-1α hydroxylation by PHD was taken from the established model of HIF-1 signaling of Nguyen et al.[2] (2013). In that model, the reaction of HIF-1α hydroxylation by PHD is described by the Michaelis-Menten kinetics, with oxygen and HIF-1α as the two substrates. We extended our model to include also HIF-2α, by adding an analogous reaction of HIF-2α hydroxylation by PHD and the corresponding ODE. We assumed that the system contains a single dominant PHD isoform, which can have potentially different activities towards HIF-1α and HIF-2α, in agreement with the available experimental evidence coming partially from ECs[3, 4]. The effects of mRNA concentrations of *HIF1A* and *EPAS1* on the translation of HIF-1α and HIF-2α and on the mRNA decay were modeled as first-order reactions using the Mass-Action Law[5, 6].

Our resulting model consists of 5 ODEs – one for each of the key molecular species participating in the response to hypoxia in HUVECs, comprising of three proteins: HIF-1α, HIF-2α, and PHD2; and two mRNAs: *HIF1A* and *EPAS1.* In our model, these molecular species participate in the reactions shown in Supplementary Information **Figure S1 A**. For each of these molecular species we had quantitative time-series data on their relative abundance at 12 time-points over 48 hours from HUVECs under 1% hypoxia, which were our published[7] and unpublished (for PHD2) data. These data are shown as points in **Figure S1 B** and are provided as **Supplementary File 1.** The changes in HIF-1α, HIF-2α and PHD2 protein levels were evaluated by Western blot densitometry and normalized to β-actin and total protein levels and expressed as a fold change over normoxic samples (0 h). *HIF1A* and *EPAS1* mRNA levels were quantified by qRT-PCR in 3 replicates, normalized to 18S mRNA levels and also expressed as a fold change over normoxic samples (0 h). We assumed normoxia was the initial condition for the model fitting, therefore the staring relative concentration of every species in our model was always 1.

After automatically fitting the ODE model to the time-series data from HUVECs over the 48 hours of 1% hypoxia, we used the fitted model to perform simulations, in which we introduced an additional drop in the oxygen level from 1% to 0.3% at 4 h from the start of hypoxia. In the results from these simulations we looked at effect that this drop had on the levels of HIF-1α and HIF-2α proteins at 8 h and 24 h of hypoxia. In other words, we used the ODE model that had been fit to the time-series data from HUVECs over the 48 hours of 1% hypoxia to predict the results of a

related experiment (not used during the initial fitting step), namely the results of a further drop in the oxygen level from 1% to 0.3% at 4 h of 1% hypoxia. Several rounds of simulation were performed, with adjustments of the model parameters from the previous round, with an aim to obtain an ODE model thereafter referred as “final” that shows not only a good fit to the time-series data from 1% hypoxia, but also a qualitative agreement between the simulation result and the experimentally observed effects of the drop in the oxygen level from 1% to 0.3% on the levels of HIF-1α and HIF-2α proteins measured at 8 h and 24 h.

As shown in **Figure S1 B**, with our final ODE model we obtained a remarkably good fit of the model to the time-series data from HUVECs over the 48 hours of 1% hypoxia. In particular, our model reflects the reduction in the *HIF1A,* but not *EPAS1* mRNA, level during hypoxia as compared to normoxia shown before (on data from an independent experiment, not used during the fitting) in Figure 6 DE. Moreover, as can be appreciated from **Figure S1 CD**, the predictions of simulation from our final ODE model are qualitatively similar to the results of the experiments shown in Figure 6BC. In particular, in the simulation and in the experiment the drop in the oxygen level from 1% to 0.3% at 4 h of hypoxia increased the concentration of HIF-1α at 8 h of hypoxia.

In our model, the oxygen level can affect the HIF-1α protein concentration only by changing the rate of HIF-1α hydroxylation by PHD. Therefore, the observed partial agreement between the model prediction (**Figure S1 CD**) and the experiment results (Figure 6 BC) provides additional illustrative support to the experimentally-derived conclusion of the current work that the residual PHD activity together with the decreasing *HIF1A* transcript levels during hypoxia contribute to the HIF-1α to HIF-2α transition.

A word of caution is in order: our ODE model is under-determined, with many different parameter values leading to similar behaviors. Therefore, our use of the modeling is mainly to provide an illustration of the proposed mechanism: how the dependencies among PHD, HIFs α, and the mRNAs encoding them could contribute to the HIF-1α to HIF-2α transition. Furthermore, the ODE model permits studying the dynamics of the described process and the effects of different timing of interventions. The full dynamic responses of our final model to 1% hypoxia and to the further drop to 0.3% oxygen at 4 h are shown in **Figure S1 EF**.

The equations (ODEs) of our model are shown in **Figure S1 G**. The full details of the model, including values of all parameters and the initial conditions, are provided in the SMBL format (https://sbml.org/) as **Supplementary File 2*.*** The model implementation, fitting and simulations were done using Matlab (MathWorks) with the SimBiology package.

A

$$\varnothing \xrightarrow{kp_{in}} \text{PHD2} \xrightarrow{kp_{out}} \varnothing$$
$$\varnothing \xrightarrow{km1_{in}} HIF1A_{mRNA} \xrightarrow{km1_{out}} \varnothing$$
$$\varnothing \xrightarrow{km2_{in}} EPAS1_{mRNA} \xrightarrow{km2_{out}} \varnothing$$
$$HIF1A_{mRNA} \overset{kt1}{\dashrightarrow} \text{HIF-1}\alpha \xrightarrow{kp1_{out}} \varnothing$$
$$EPAS1_{mRNA} \overset{kt2}{\dashrightarrow} \text{HIF-2}\alpha \xrightarrow{kp2_{out}} \varnothing$$
$$\text{HIF-1}\alpha + O_2 \xrightarrow{\text{PHD2}} \varnothing$$
$$\text{HIF-2}\alpha + O_2 \xrightarrow{\text{PHD2}} \varnothing$$

B

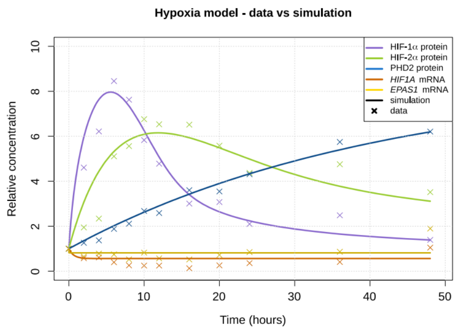

C **D** **E** **F**

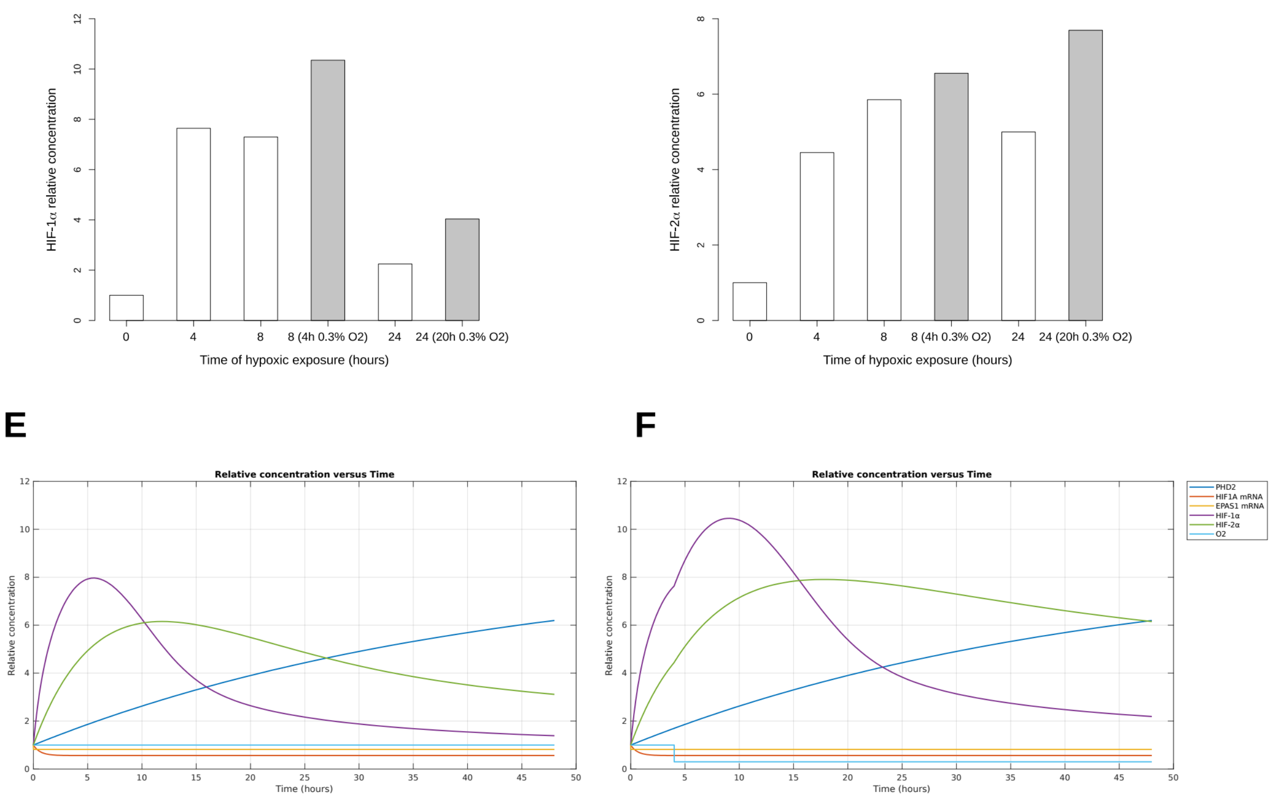

G

$$\frac{d[\text{PHD2}](t)}{dt} = kp_{in} - kp_{out}[\text{PHD2}](t)$$
$$\frac{d[HIF1A_{mRNA}](t)}{dt} = km1_{in} - km1_{out}[HIF1A_{mRNA}](t)$$
$$\frac{d[EPAS1_{mRNA}](t)}{dt} = km2_{in} - km2_{out}[EPAS1_{mRNA}](t)$$
$$\frac{d[\text{HIF-1}\alpha](t)}{dt} = -\frac{Vm1 * O_2}{km1+O_2}\frac{[\text{PHD2}](t)*[\text{HIF-1}\alpha](t)}{nm1+[\text{HIF-1}\alpha](t)} + kt1[HIF1A_{mRNA}](t) + kp1_{in} - kp1_{out}[\text{HIF-1}\alpha](t)$$
$$\frac{d[\text{HIF-2}\alpha](t)}{dt} = -\frac{Vm2 * O_2}{km2+O_2}\frac{[\text{PHD2}](t)*[\text{HIF-2}\alpha](t)}{nm2+[\text{HIF-2}\alpha](t)} + kt2[EPAS1_{mRNA}](t) + kp2_{in} - kp2_{out}[\text{HIF-2}\alpha](t)$$

Supplementary Figure S1. A dynamic model of regulation of HIF-1α and HIF-2α protein levels. The time 0 represents normoxia, which was the initial condition during fitting and simulations. (A) The model reactions. (B) The time-series data from HUVECs over the 48 hours of 1% hypoxia (points) and the evolution of the final ODE model fitted to these data (continuous lines). (C, D) The

predicted response of the final ODE model to a further drop in the oxygen level to 0.3% oxygen at 4 h of hypoxia for HIF-1α (C) and HIF-2α (D). (E) The full response of the final ODE model to 1% oxygen hypoxia (fitted values repeated from panel B drawn on different scale for comparison with panel F). (F). The full response of the final ODE model to 1% oxygen hypoxia and a further drop to 0.3% oxygen at 4 h (predictions). (G) The equations (ODEs) of the model.

References

1. Cavadas MA, Nguyen LK, Cheong A. Hypoxia-inducible factor (HIF) network: insights from mathematical models. *Cell Commun Signal* **11**:42 (2013).

2. Nguyen LK, *et al.* A dynamic model of the hypoxia-inducible factor 1α (HIF-1α) network. *J Cell Sci* **126**:1454–1463 (2013).

3. Appelhoff RJ, *et al.* Differential function of the prolyl hydroxylases PHD1, PHD2, and PHD3 in the regulation of hypoxia-inducible factor. *J Biol Chem* **279**:38458–38465 (2004).

4. Cioffi CL, Liu XQ, Kosinski PA, Garay M, Bowen BR. Differential regulation of HIF-1 alpha prolyl-4-hydroxylase genes by hypoxia in human cardiovascular cells. *Biochem Biophys Res Commun* **303**:947–953 (2003).

5. Lund EW. Guldberg and Waage and the law of mass action. *J Chem Educ* **42**:548 (1965).

6. Érdi P, Tóth J. Mathematical Models of Chemical Reactions: Theory and Applications of Deterministic and Stochastic Models */ P. Érdi and J. Tóth*. Manchester University Press (1989).

7. Bartoszewski R, *et al.* Primary endothelial cell-specific regulation of hypoxia-inducible factor (HIF)-1 and HIF-2 and their target gene expression profiles during hypoxia. *FASEB J* **33**:7929–7941 (2019).
