# Supplementary material for: The transition from HIF-1 to HIF-2 during prolonged hypoxia results from reactivation of PHDs and HIF1A mRNA instability
Source: Cell Mol Biol Lett. 2022 Dec 8;27:109. doi: 10.1186/s11658-022-00408-7 (PMC9730601; doi:10.1186/s11658-022-00408-7)

Supplemental material for **Figure 1** *– uncropped Western blots*

Replicates 1 and 2

HIF-1α

Sample Set 1 | Sample Set 2

Time of hypoxic exposure (hours): 0 4 8 24 0 4 8 24

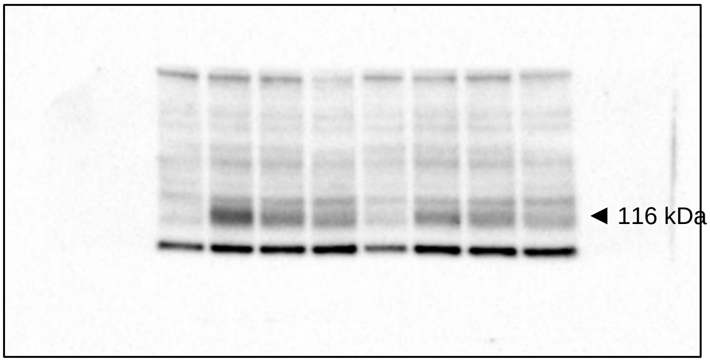

◄ 116 kDa

HIF-2α

Sample Set 1 | Sample Set 2

Time of hypoxic exposure (hours): 0 4 8 24 0 4 8 24

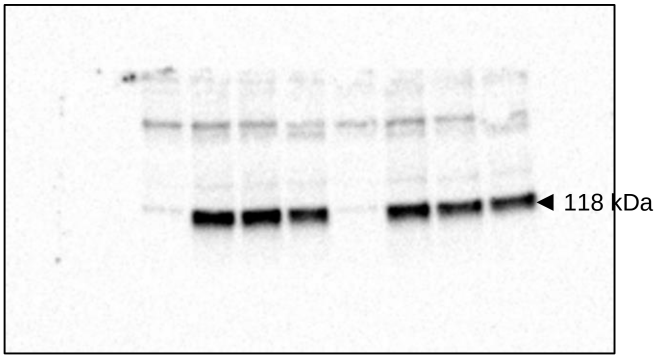

◄ 118 kDa

Total protein

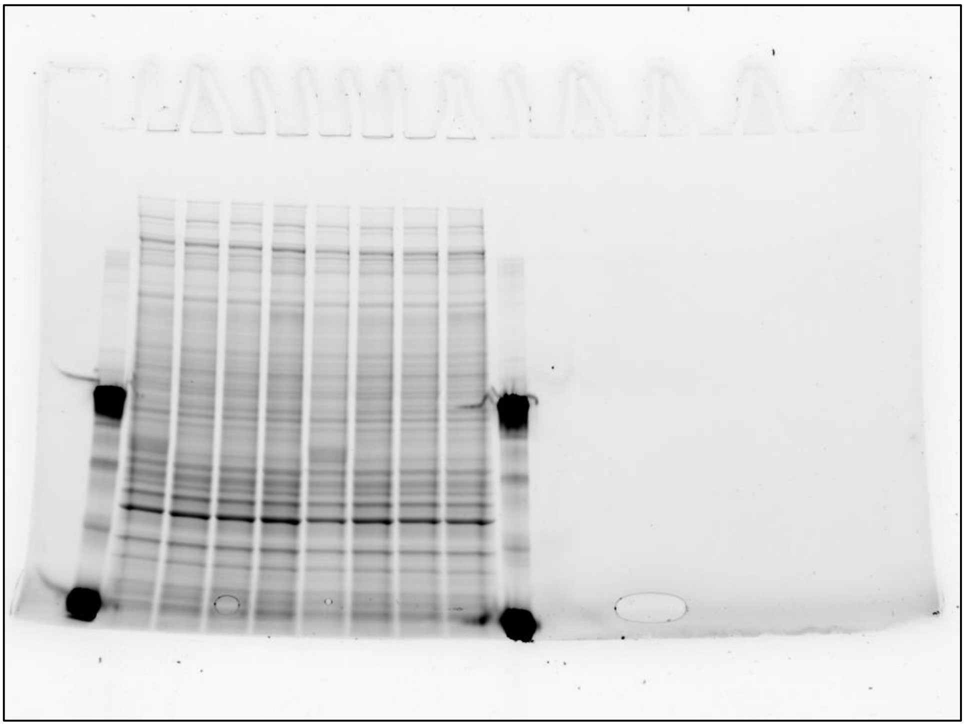

Supplemental material for Figure 1 – *uncropped Western blots*

β-actin

Replicates 1 and 2

Sample Set 1 | Sample Set 2

Time of hypoxic exposure (hours): 0 4 8 24 0 4 8 24

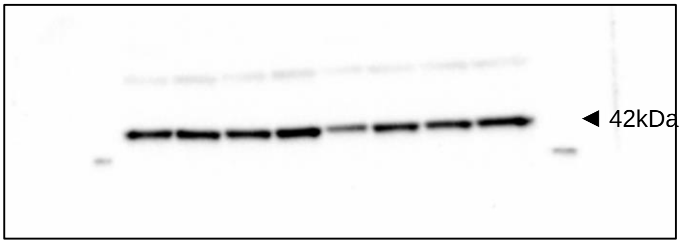

◄ 42kDa

Replicate 3

HIF-1α

Sample Set 1

Time of hypoxic exposure (hours): 0 4 8 24

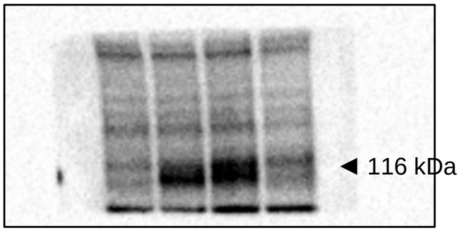

◄ 116 kDa

HIF-2α

Sample Set 1

Time of hypoxic exposure (hours): 0 4 8 24

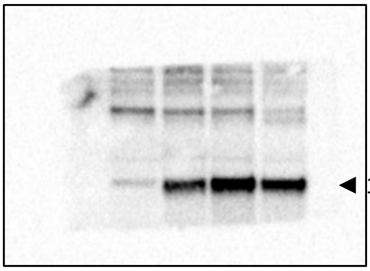

◄ 118 kDa

β-actin

Sample Set HIF-1α | Sample Set HIF-2α

Time of hypoxic exposure (hours): 0 4 8 24 0 4 8 24

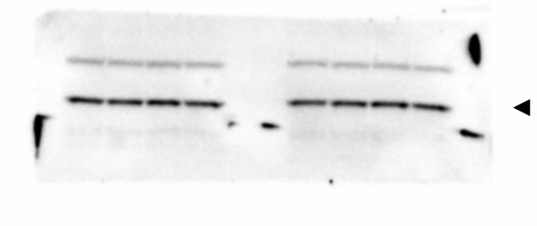

◄ 42kDa

Supplemental material for Figure 1 – *uncropped Western blots*

Replicate 3

Total protein

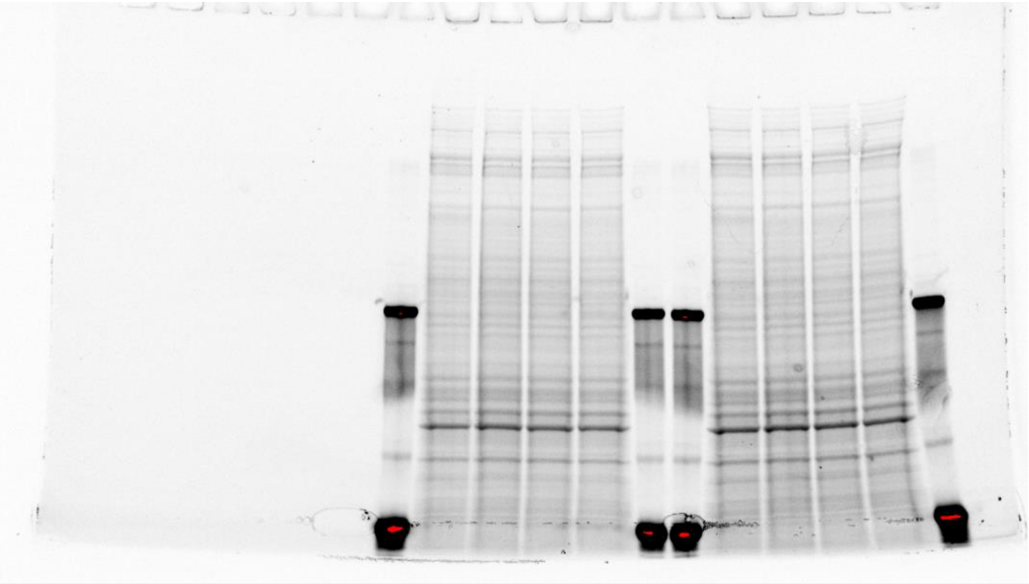

Replicate 4

HIF-1α

Sample Set 1

Time of hypoxic exposure (hours): 0 4 8 24

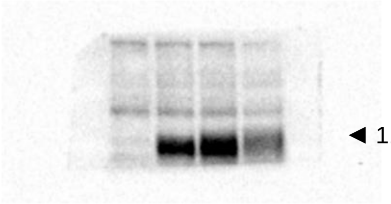

◀ 116 kDa

HIF-2α

Sample Set 1

Time of hypoxic exposure (hours): 0 4 8 24

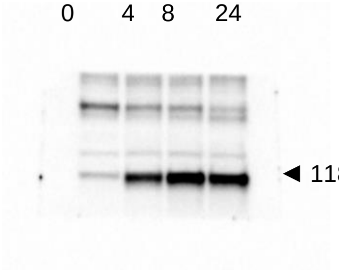

◀ 118 kDa

β-actin

Sample Set HIF-1α | Sample Set HIF-2α

Time of hypoxic exposure (hours): 0 4 8 24 | 0 4 8 24

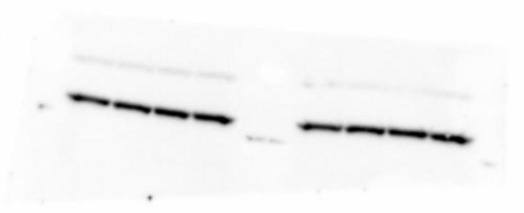

◀ 42kDa

Supplemental material for Figure 1 – *uncropped Western blots*

Replicate 4

Total protein

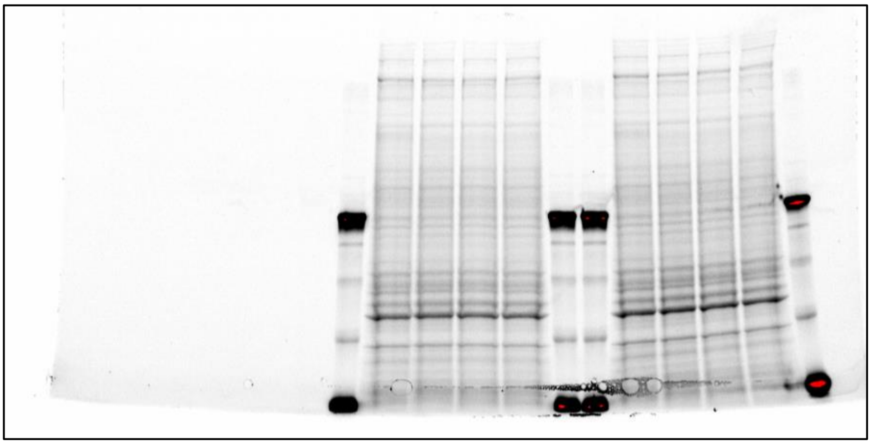

Replicate 5&6

HIF-1α

Time of hypoxic exposure (hours)

Sample Set 1 Sample Set 2

0 4 8 24 0 4 8 24

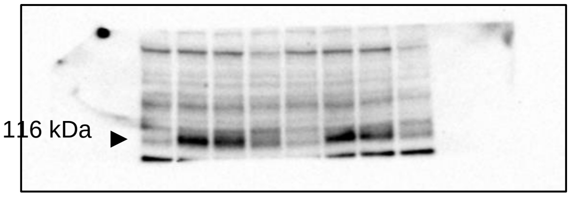

116 kDa ▶

HIF-2α

Sample Set 1 Sample Set 2

0 4 8 24 0 4 8 24

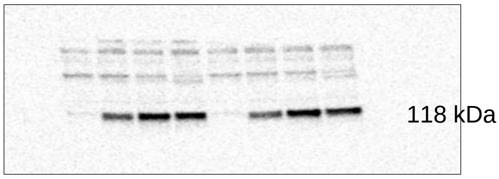

118 kDa

Total protein

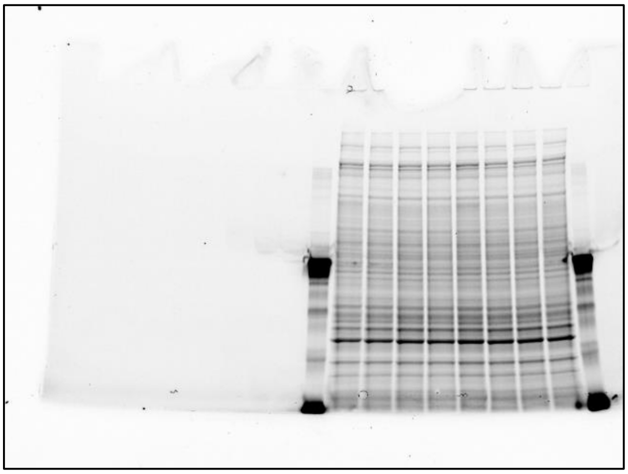

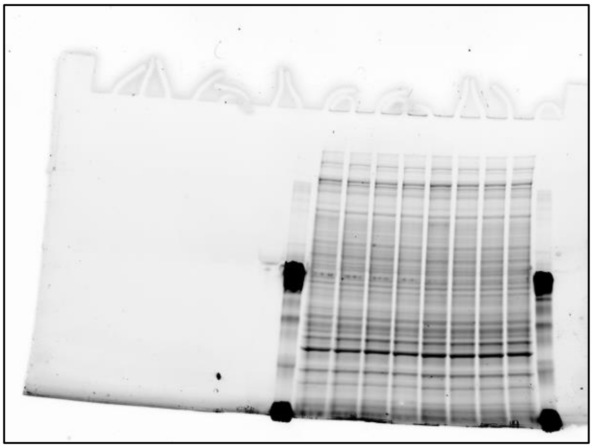

Supplemental material for Figure 2 – *uncropped Western blots*

Replicate 1

HIF-1α

Time of hypoxic exposure (hours):	0	4				8				16			
Cyclohexmide treatment (minutes)	0	0	20	45	90	0	20	45	90	0	20	45	90

116 kDa

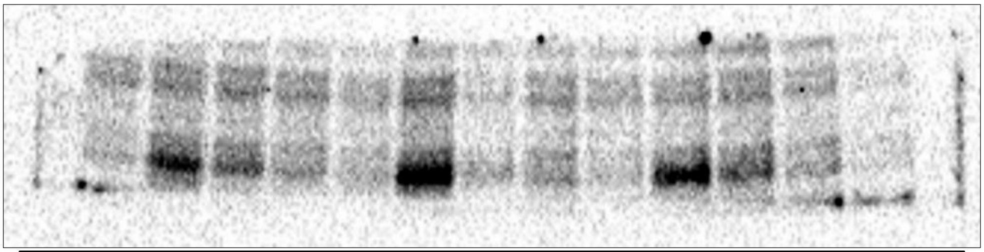

Total protein

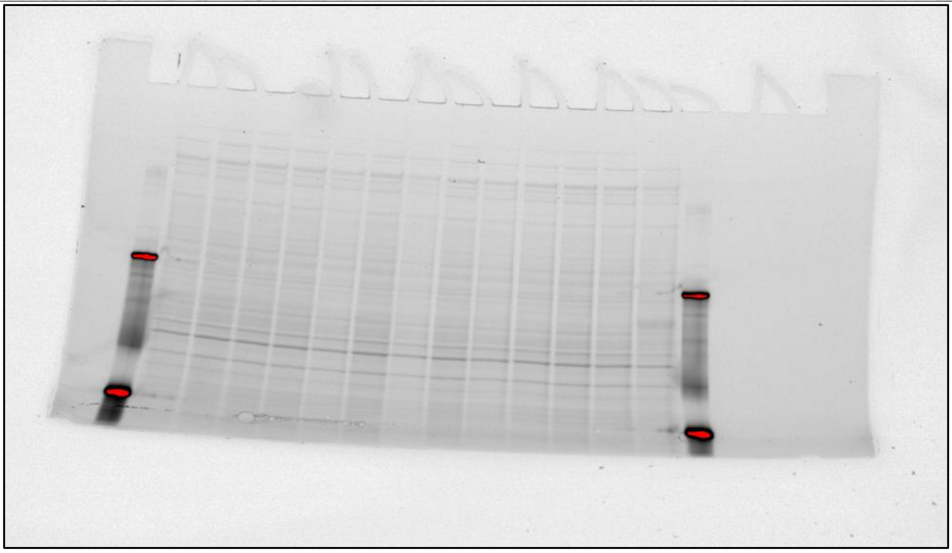

Replicate 2

HIF-1α

Time of hypoxic exposure (hours):	0	4				8				16			
Cyclohexmide treatment (minutes)	0	0	20	45	90	0	20	45	90	0	20	45	90

116 kDa

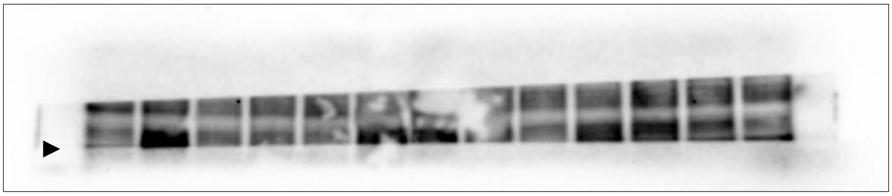

Total protein

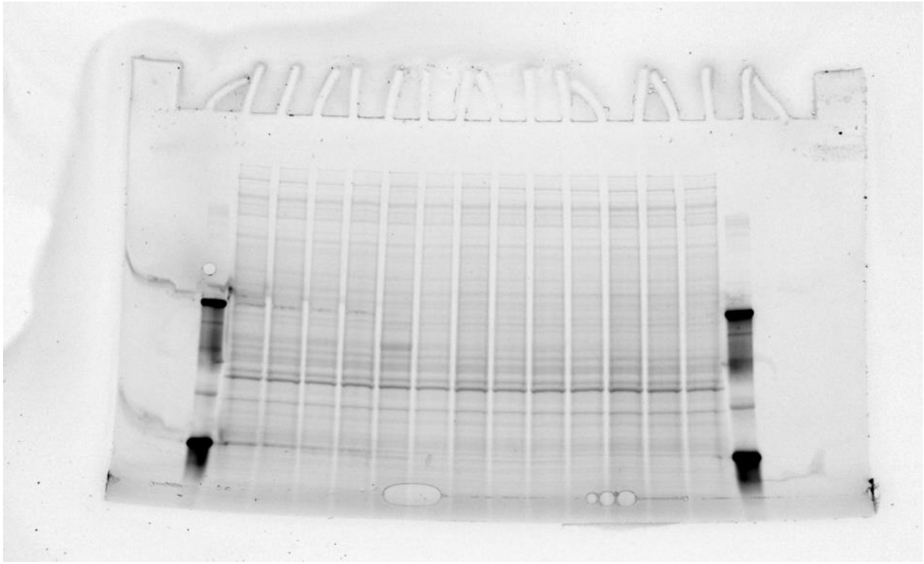

Supplemental material for Figure 2 *– uncropped Western blots*

Replicate 3

HIF-1α

Time of hypoxic exposure (hours): 0 | 4 | 8 | 16

Cyclohexmide treatment (minutes) 0 | 0 20 45 90 | 0 20 45 90 | 0 20 45 90

116 kDa ▶

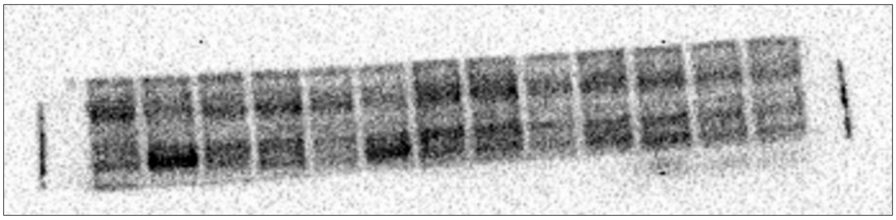

Total protein

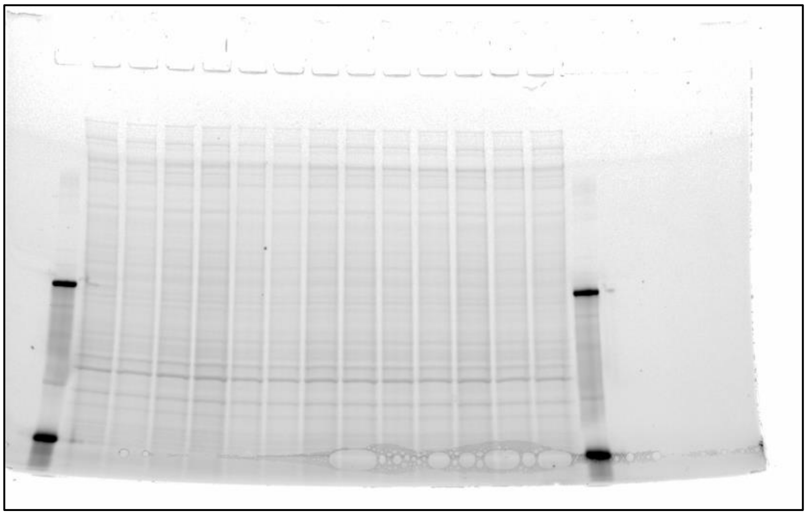

Replicate 4

HIF-1α

Time of hypoxic exposure (hours): 0 | 4 | 8 | 16

Cyclohexmide treatment (minutes) 0 | 0 20 45 90 | 0 20 45 90 | 0 20 45 90

116 kDa ▶

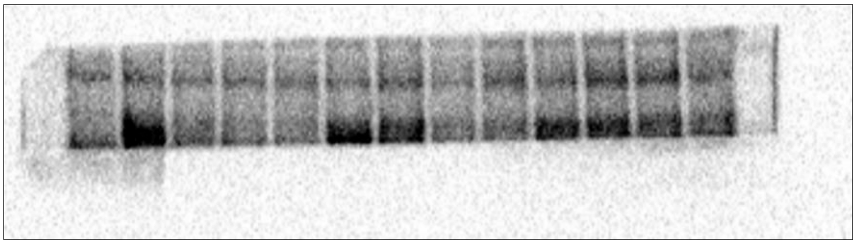

Total protein

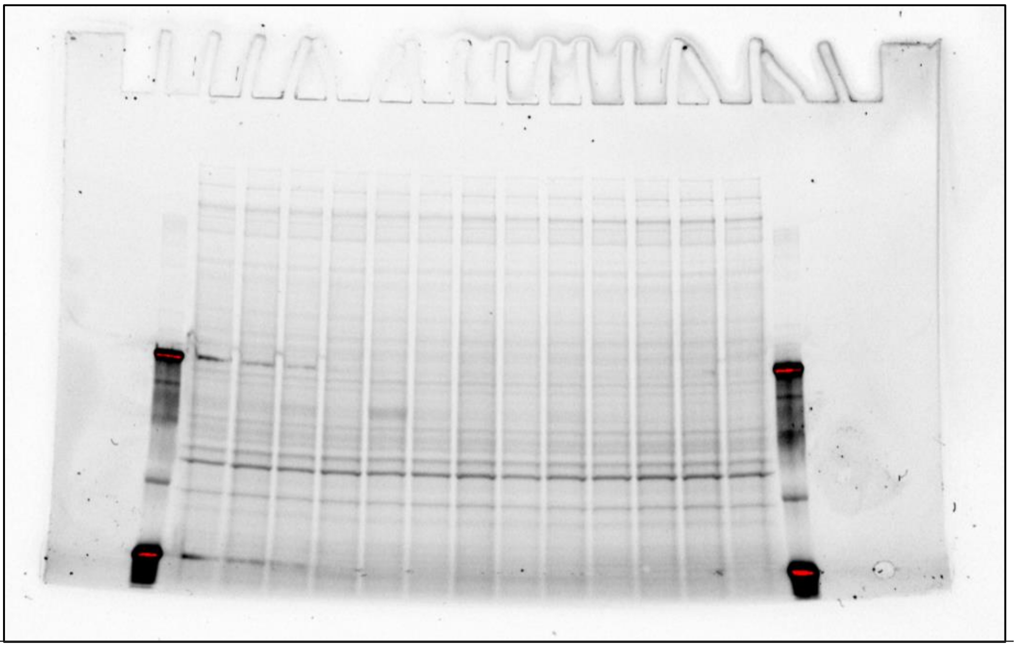

Supplemental material for Figure 3 – *uncropped Western blots*

Replicate 1

HIF-2α

Time of hypoxic exposure (hours): 0 | 4 | 8 | 16

Cyclohexmide treatment (minutes) 0 | 0 20 45 90 | 0 20 45 90 | 0 20 45 90

118 kDa ►

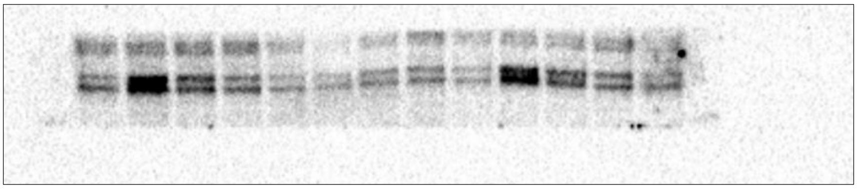

Total protein

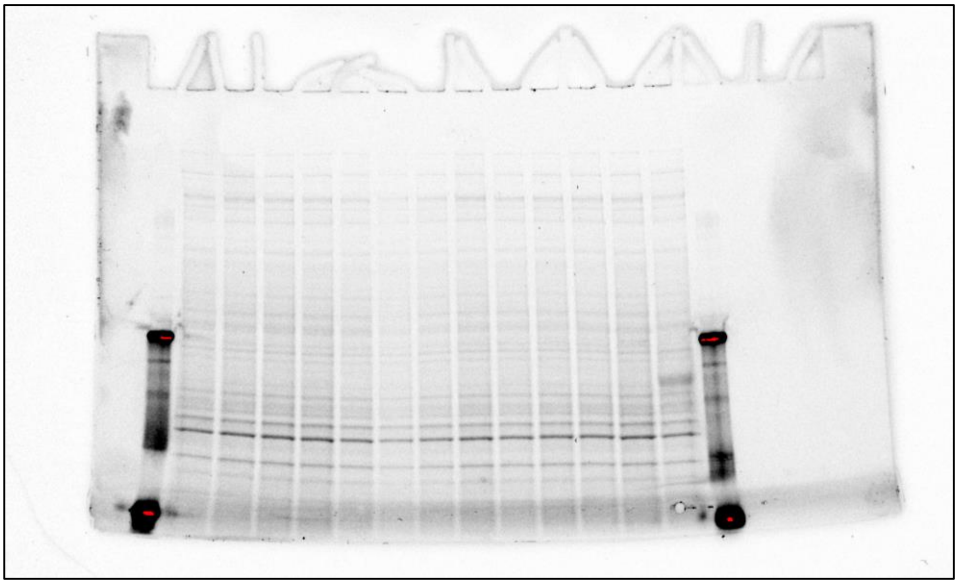

Replicate 2

HIF-2α

Time of hypoxic exposure (hours): 0 | 4 | 8 | 16

Cyclohexmide treatment (minutes) 0 | 0 20 45 90 | 0 20 45 90 | 0 20 45 90

118 kDa ►

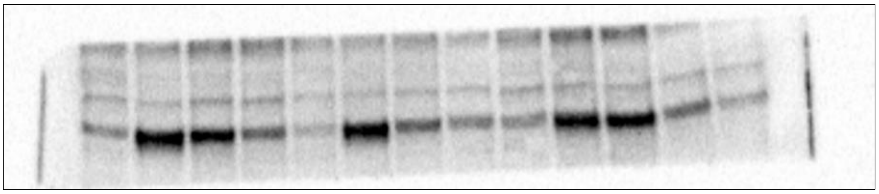

Total protein

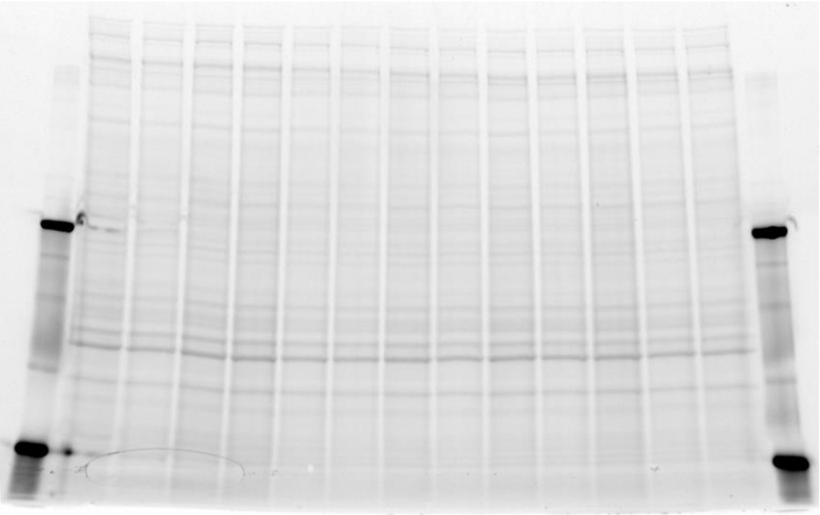

Supplemental material for Figure 3 – *uncropped Western blots*

Replicate 3

HIF-2α

Time of hypoxic exposure (hours):	0	4				8				16			
Cyclohexmide treatment (minutes)	0	0	20	45	90	0	20	45	90	0	20	45	90

118 kDa ▶

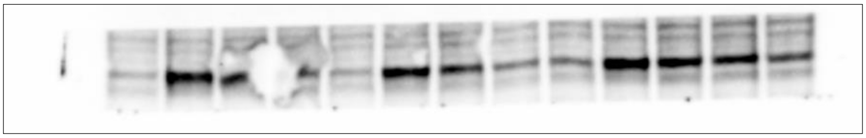

Total protein

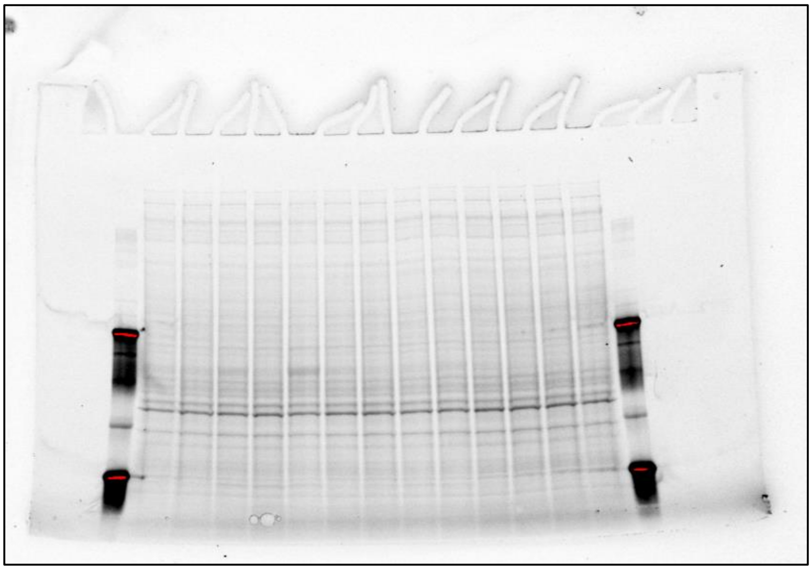

Replicate 4

HIF-2α

Time of hypoxic exposure (hours):	0	4				8				16			
Cyclohexmide treatment (minutes)	0	0	20	45	90	0	20	45	90	0	20	45	90

118 kDa ▶

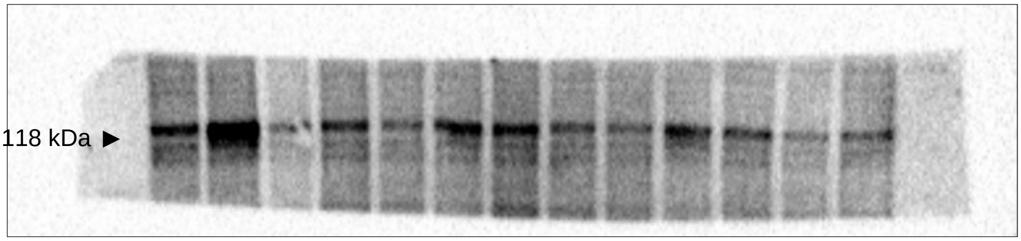

Total protein

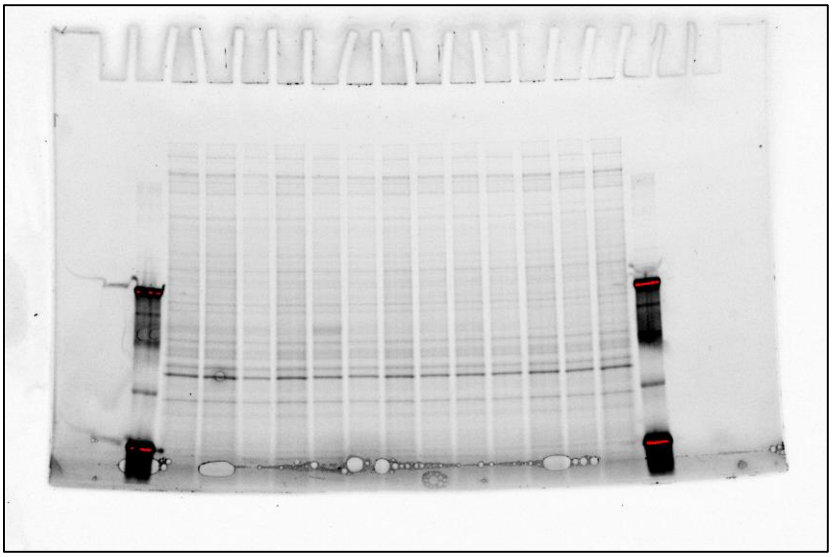

Supplemental material for Figure 3 – *uncropped Western blots*

Replicate 5

HIF-2α

Time of hypoxic exposure (hours): 0 | 4 | 8 | 16

Cyclohexmide treatment (minutes) 0 | 0 20 45 90 | 0 20 45 90 | 0 20 45 90

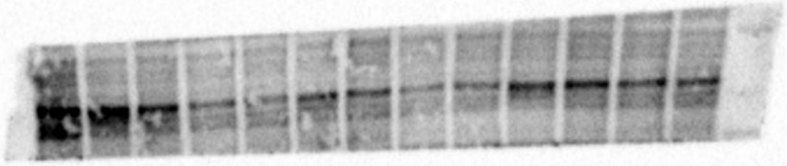

118 kDa ▶

Total protein

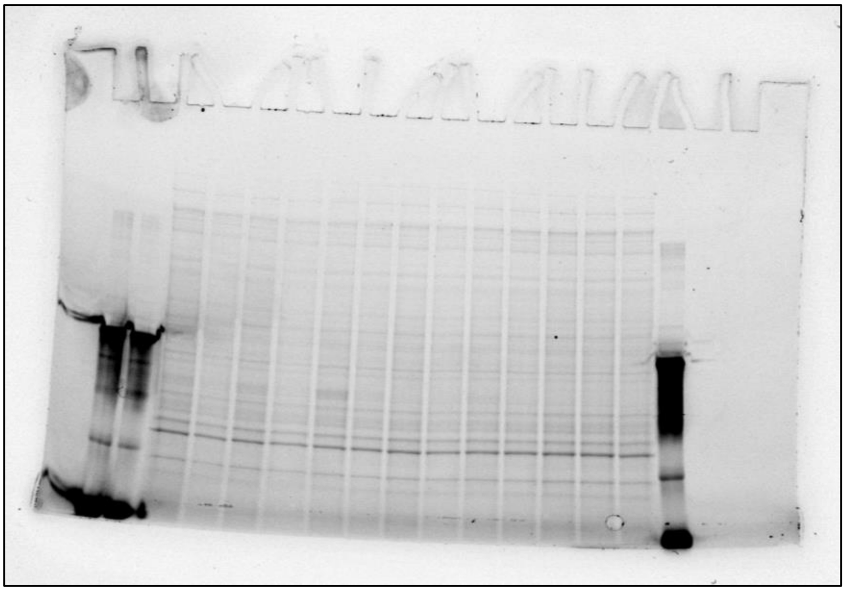

Supplemental material for Figure 5 – *uncropped Western blots*

Replicate 1

HIF-1α

Time of hypoxic exposure (hours)	0	4	8	8	24	24
DMOG treatment (hours)	0	0	0	**4**	0	**20**

116 kDa ►

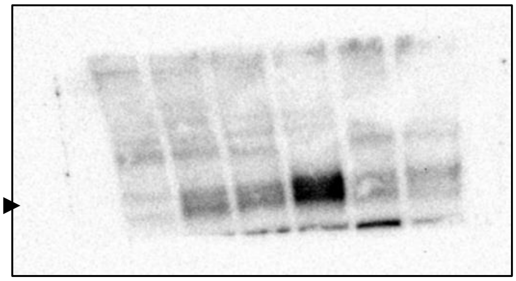

HIF-2α

0	4	8	8	24	24
0	0	0	**4**	0	**20**

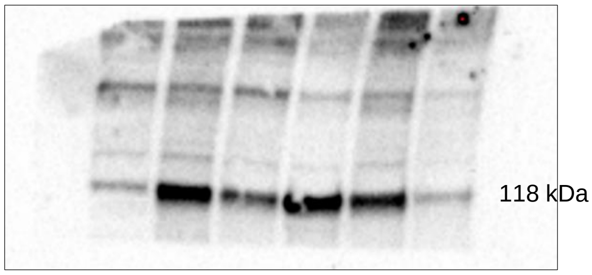

118 kDa

Total protein

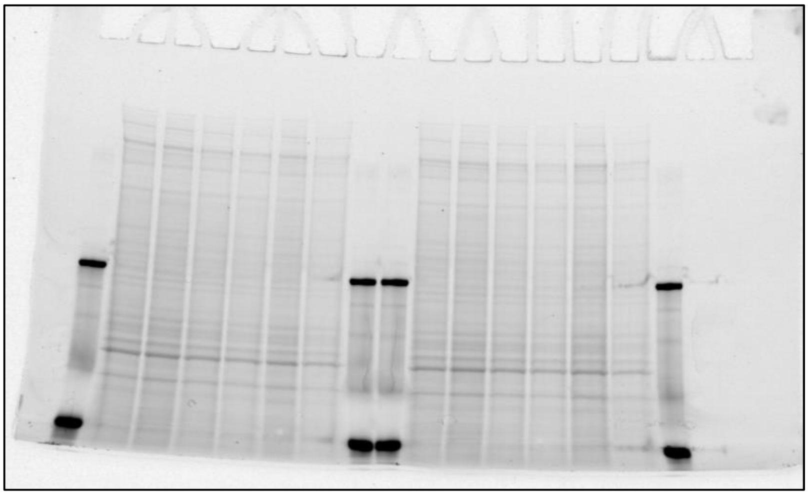

Replicate 2

HIF-1α

Time of hypoxic exposure (hours)	0	4	8	8	24	24
DMOG treatment (hours)	0	0	0	**4**	0	**20**

116 kDa ►

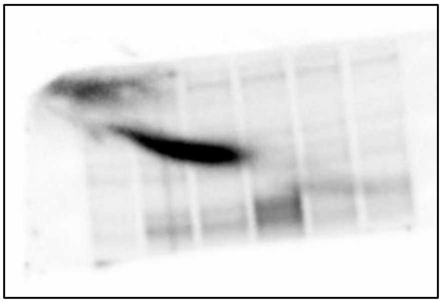

HIF-2α

0	4	8	8	24	24
0	0	0	**4**	0	**20**

►

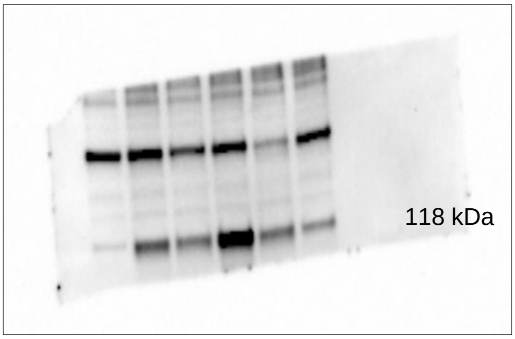

Supplemental material for Figure 5 – *uncropped Western blots*

Replicate 2

Total protein

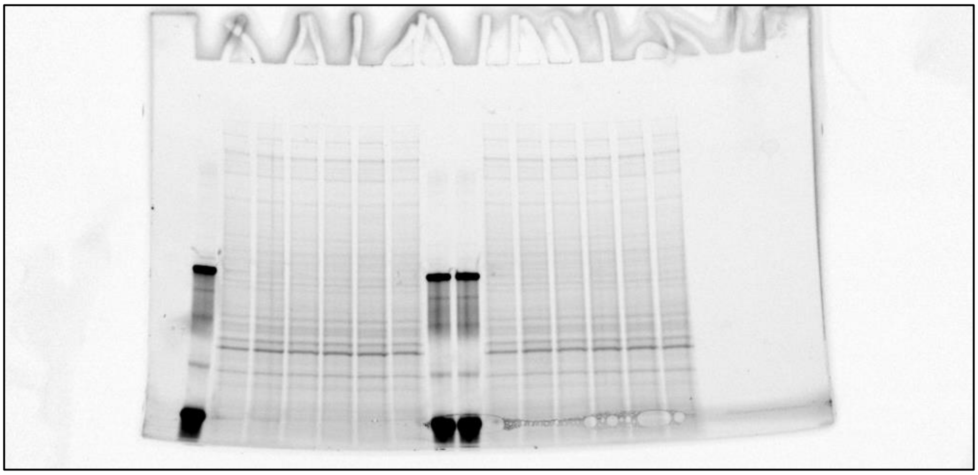

Replicate 3

HIF-1α

Time of hypoxic exposure (hours)	0	4	8	8	24	24
DMOG treatment (hours)	0	0	0	**4**	0	**20**

116 kDa ▶

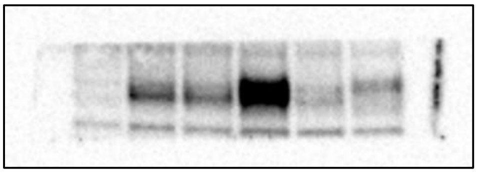

HIF-2α

0	4	8	8	24	24
0	0	0	**4**	0	**20**

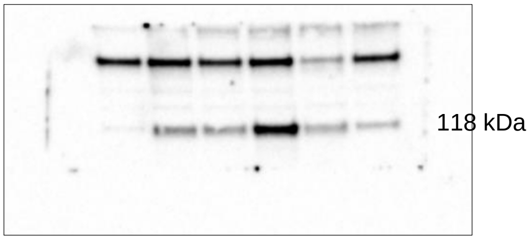

118 kDa

Total protein

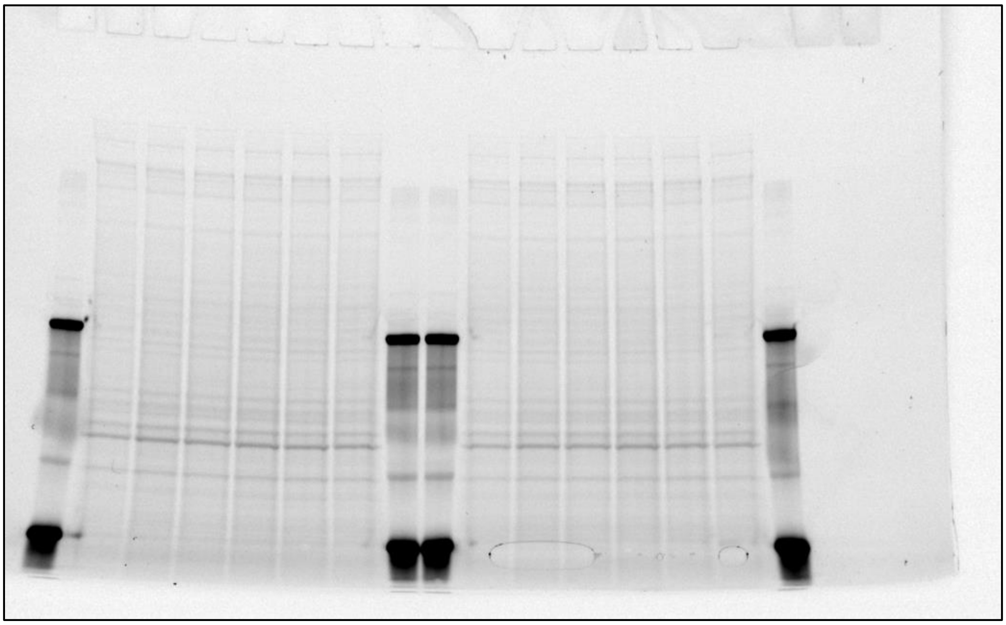

Supplemental material for Figure 5 – *uncropped Western blots*

Replicates 4 & 5

HIF-2α

Time of hypoxic exposure (hours)	0	4	8	8	24	24
DMOG treatment (hours)	0	0	0	**4**	0	**20**

118 kDa ▶

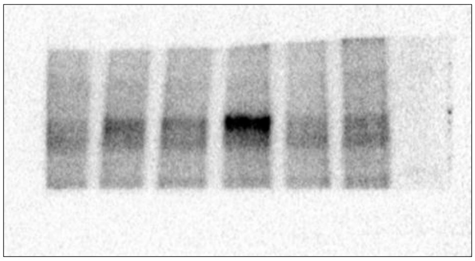

HIF-2α

0	4	8	8	24	24
0	0	0	**4**	0	**20**

118 kDa

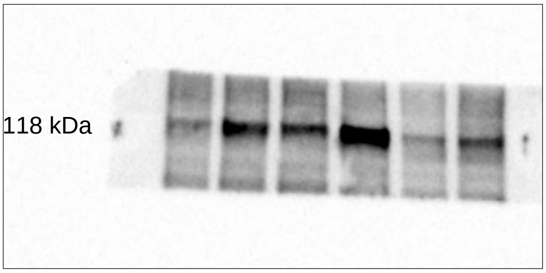

Total protein

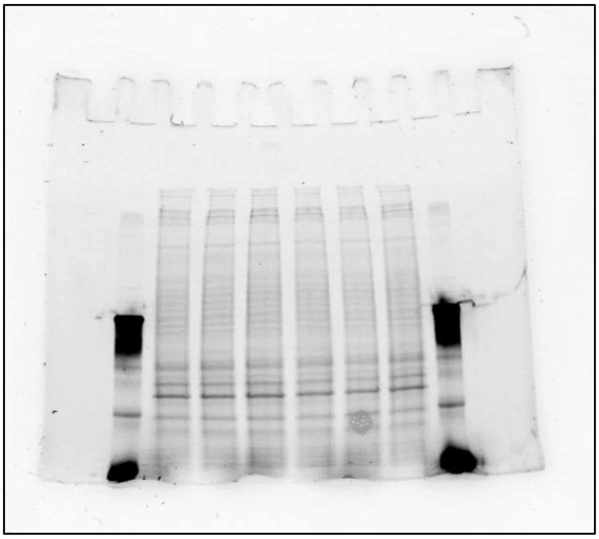

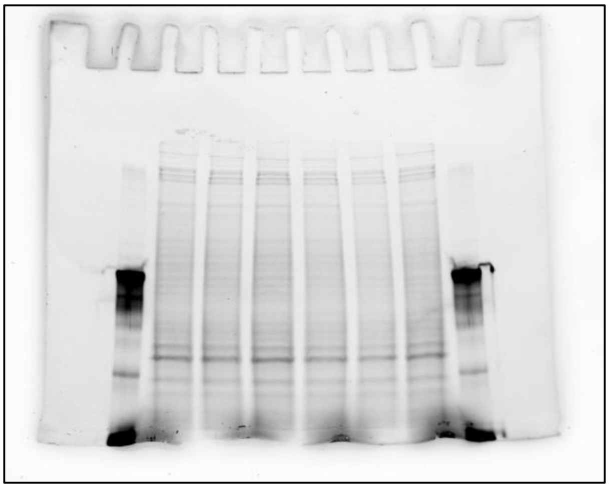

Supplemental material for Figure 6 – *uncropped Western blots*

Replicate 1

HIF-1α

Time of hypoxic exposure (hours)	0	4	8	8	24	24
Time of 0.3% O_2 exposure (hours)	0	0	0	**4**	0	**20**

116 kDa ►

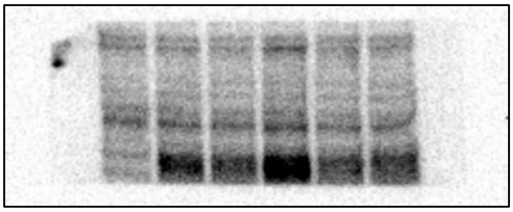

Total protein

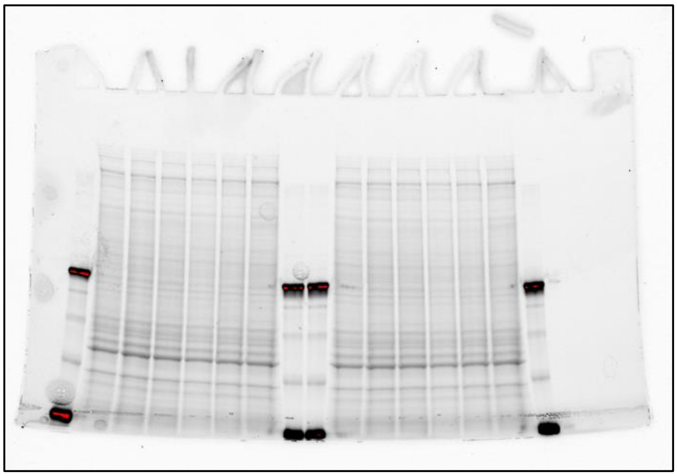

Replicate 2

HIF-1α

Time of hypoxic exposure (hours)	0	4	8	8	24	24
Time of 0.3% O_2 exposure (hours)	0	0	0	**4**	0	**20**

116 kDa ►

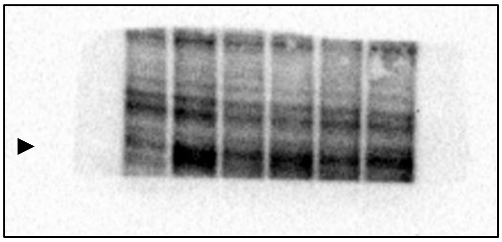

HIF-2α

0	4	8	8	24	24
0	0	0	**4**	0	**20**

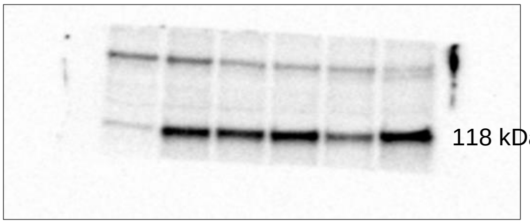

118 kDa

Total protein

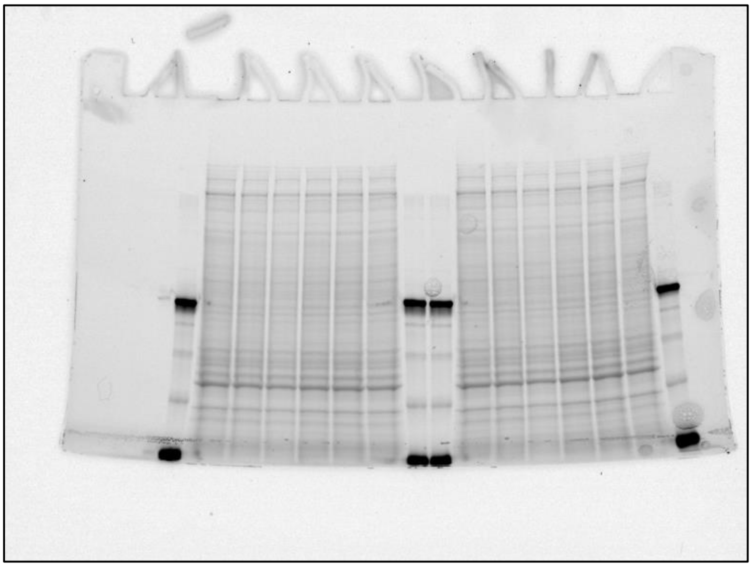

Supplemental material for Figure 6 – *uncropped Western blots*

Replicate 3

HIF-1α

Time of hypoxic exposure (hours)	0	4	8	8	24	24
Time of 0.3% O_2 exposure (hours)	0	0	0	**4**	0	**20**

116 kDa ▶

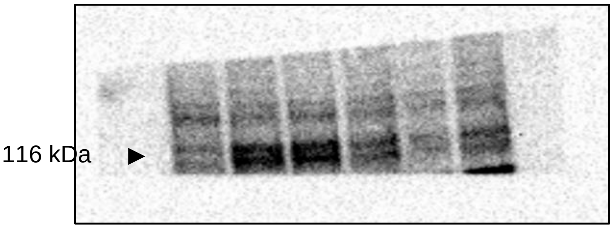

HIF-2α

	0	4	8	8	24	24
	0	0	0	**4**	0	**20**

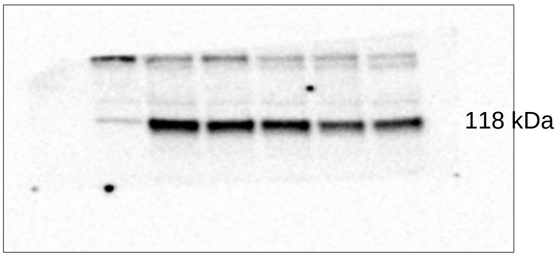

118 kDa

Total protein

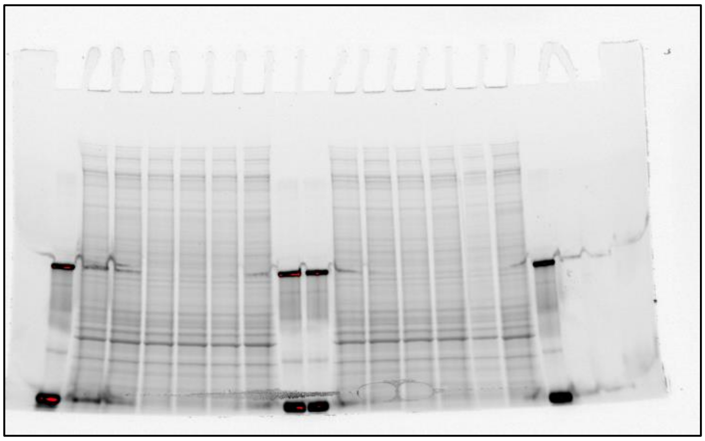

Replicate 4

HIF-2α

Time of hypoxic exposure (hours)	0	4	8	8	24	24
Time of 0.3% O_2 exposure (hours)	0	0	0	**4**	0	**20**

118 kDa ▶

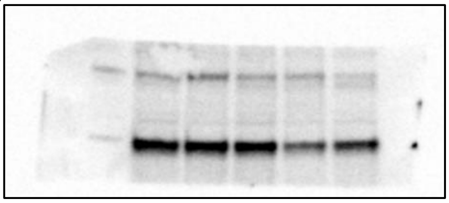

Total protein

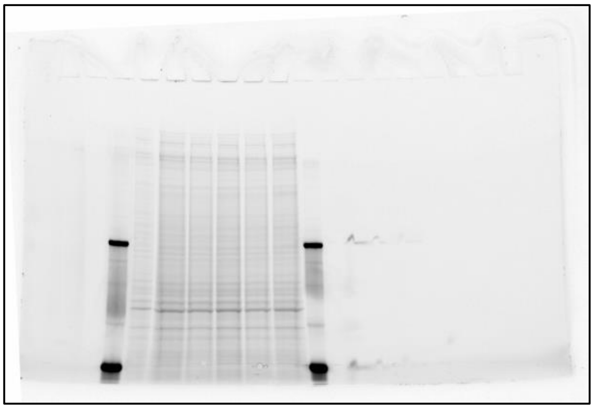

Supplemental material for Figure 6 – *uncropped Western blots*

Replicate 5

HIF-1α

Time of hypoxic exposure (hours)	0	4	8	8	24	24
Time of 0.3% O_2 exposure (hours)	0	0	0	**4**	0	**20**

116 kDa ►

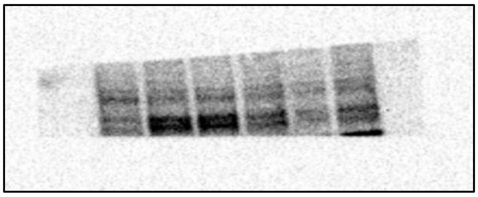

HIF-2α

Time of hypoxic exposure (hours)	0	4	8	8	24	24
Time of 0.3% O_2 exposure (hours)	0	0	0	**4**	0	**20**

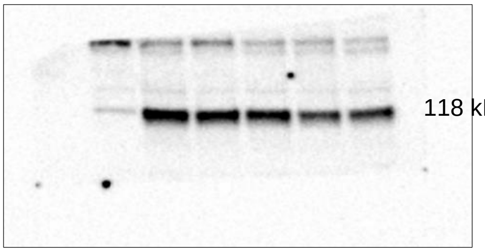

118 kDa

Total protein

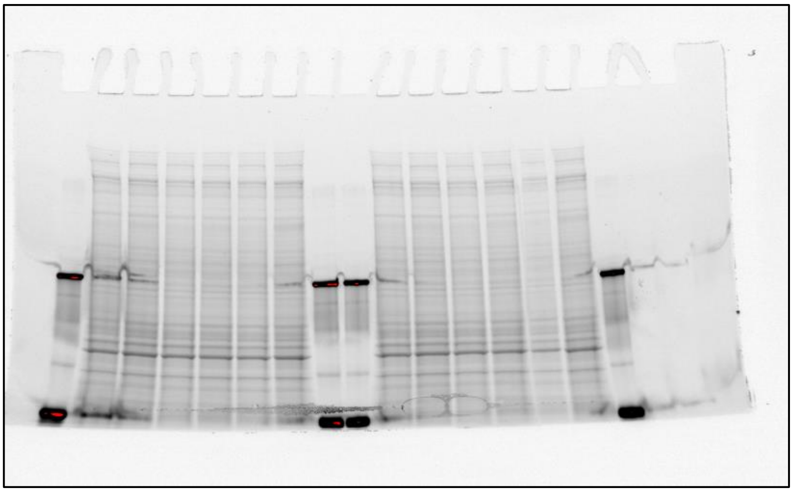

Supplement: Supplementary file 6 — Additional file 6. Supplementary Materials - western blots. [file 11658_2022_408_MOESM6_ESM.pdf]
